# The CAR macrophage cells, a novel generation of chimeric antigen-based approach against solid tumors

**DOI:** 10.1186/s40364-023-00537-x

**Published:** 2023-11-28

**Authors:** Kaveh Hadiloo, Siavash Taremi, Mahmood Heidari, Abdolreza Esmaeilzadeh

**Affiliations:** 1 Student Research Committee, School of Medicine, Zanjan University of Medical Sciences, Department of Immunology, Zanjan, Iran; 2 School of Medicine, Zanjan University of Medical Sciences, Zanjan, Iran; 3 Department of Immunology, Zanjan University of Medical Sciences, Zanjan, Iran; 4 Cancer Gene Therapy Research Center (CGRC), Zanjan University of Medical Sciences, Zanjan, Iran

**Keywords:** Adoptive cell therapy, Macrophage cell, Chimeric antigen receptor, Cancer, Challenges, Solution

## Abstract

Today, adoptive cell therapy has many successes in cancer therapy, and this subject is brilliant in using chimeric antigen receptor T cells. The CAR T cell therapy, with its FDA-approved drugs, could treat several types of hematological malignancies and thus be very attractive for treating solid cancer. Unfortunately, the CAR T cell cannot be very functional in solid cancers due to its unique features. This treatment method has several harmful adverse effects that limit their applications, so novel treatments must use new cells like NK cells, NKT cells, and macrophage cells. Among these cells, the CAR macrophage cells, due to their brilliant innate features, are more attractive for solid tumor therapy and seem to be a better candidate for the prior treatment methods. The CAR macrophage cells have vital roles in the tumor microenvironment and, with their direct effect, can eliminate tumor cells efficiently. In addition, the CAR macrophage cells, due to being a part of the innate immune system, attended the tumor sites. With the high infiltration, their therapy modulations are more effective. This review investigates the last achievements in CAR-macrophage cells and the future of this immunotherapy treatment method.

**Supplementary Information:**

The online version contains supplementary material available at 10.1186/s40364-023-00537-x.

## Introduction

Immunotherapy has come to fruition in recent years for cancer treatment by manipulating the immune system. Among the various forms of immunotherapy, adoptive cell therapy (ACT) is a brilliant and efficient way due to the tread more personalized and targeted treatments [1]. As a subset of ACT, the chimeric antigen receptor (CAR) T cells are frontier with distinctive outcomes in cancer therapy. Until now introduced, six FDA-approved drugs utilizing CAR T against hematological malignancies were introduced: Kymriah, Yescarta, Tecartus, Breyanzi, Abecma, and Carvykti [2].

However, CAR-T cell therapy has several obstacles the high cost and time-consuming production, cytokine release syndrome (CRS), immune effector cell-associated neurotoxicity syndrome (ICANS), “on-target/off-tumor” toxicity, and graft versus host disease (GVHD) [3, 4]. Indeed, the CAR T cell had less effectiveness in solid cancers due to special features like heterogeneity, antigen escaping, limited T cell fitness, inefficient homing and infiltration, and high complexity of tumor microenvironment (TME) [5]. TME has a crucial role in cancer progression and reduces the efficacy of the CAR T cells by several mechanisms such as physical barriers, rivalry for metabolic fuels, cell exhaustion, immunosuppressive microenvironment, and various immunosuppressive cells like T regulatory (Treg) cells, myeloid-derived suppressor cells (MDSCs), and tumor-associated macrophages (TAMs) [6, 7]. TAMs have the highest infiltration rate among immune cells in TME. Most possess pro-tumoral and immunosuppressive (M2-like phenotype) states that promote angiogenesis tumor invasion and aid in metastasis [8, 9]. However, because of the plasticity feature of macrophages, it can be reversed to an anti-tumor (M1) phenotype with the potency of phagocytosis, antigen presentation, and inflammatory cytokines secretion [10]. In past studies, the macrophage cells were used by different methods versus cancers which acquired significant results in the laboratory, while the clinical trials did not achieve substantial results, so novel improvement methods are needed to create potent macrophages [11, 12].

The CAR macrophage (CAR M) cells are created by engineering macrophage cells to express CAR. Utilizing CAR M cells as a novel therapeutic method could be a ray of hope to overcome the challenges of solid tumors. In this review, we explained the macrophage cell and their roles in TME, correlation between the CAR structure and these cells, compared CAR M cells to other CAR-armored cells, introduced the CAR M cell as a promising candidate for solid cancer therapies, discussed the challenges, and in the end, offered some solutions to create effective anti-cancer cells.

### The macrophage cells are the living director in correlation with cancer cells

Macrophages, as innate immune cells, participate in immune defense, tissue homeostasis, and regulation of diseases [11, 13]. The infiltration rate of macrophages based on their heterogenicity had various effects on the prognosis of cancers. In most cancers, the reverse correlation exists between the macrophage infiltration rate and prognosis (like in bladder, glioma, breast, melanoma, and prostate). Still, this correlation is entirely directed in some cancers seen, such as gastric (GC) and colorectal cancers [14–20].

Traditionally, the macrophage has two main phenotypes, including the “classically activated macrophages” (M1) and “alternatively activated macrophages” (M2) types. However, the M1/M2 dichotomy is a simplified model, and these cells have an extreme spectrum of M1 to M2 phenotypes [21]. Macrophages undergo M1 polarization by exposing to interleukin (IL)-12, tumor necrosis factor-α (TNFα), interferon-gamma (IFNγ), granulocyte–macrophage colony-stimulating factor (GM-CSF), and bacterial lipopolysaccharide (LPS). M1 macrophages are identified by the expression of CD68, CD80, CD86, major histocompatibility complex II (MHCII), and inducible nitric oxide synthase (iNOS). These cells have anti-tumor and pathogen-killing capabilities, reactive oxygen species secreting, higher antigen-presenting ability, proinflammatory cytokine production, and play a part in T-helper (Th) type 1 response. Macrophages polarize to M2 phenotype by being exposed to IL-4, 5, 10, 13, colony-stimulating factor 1 (CSF1), transforming growth factor-beta (TFG-β), and prostaglandin E_2_ (PGE_2_). The M2 macrophages determine by the expression of CD206, CD204, vascular endothelial growth factor (VEGF), CD163, and arginase-1 (Arg-1). M2 macrophage exhibits pro-tumoral, anti-inflammatory, tissue repair and remodeling, secrete cytokines like IL-6, IL-10, and TGF-β, and is involved in Th type 2 response [22–24]. The M1 and M2 macrophage have distinct metabolism; M1 depends on glycolytic metabolism, while M2 depend on glutamine consumption, fatty acids oxidation, and the tricarboxylic acid cycle [24]. Macrophages are recruited in tumor sites by various chemokines such as CSF-1, CCL2, 5, CXCL8, and 12, which are secreted from TME. Also, IL-4, 6, 10, CCL2, 5, CSF-1, TGF-β, lactic acid accumulation, and tumor-derived exosomes (TEXs) promote M2 polarization in TAM cells[25–27].

TAMs are derived from MDSCs, bone marrow (BM)-derived monocytes, and tissue-resident macrophages. TAMs have heterogeneous features that extend from pro-tumoral to anti-tumoral. However, most TAMs possess pro-tumoral characteristics in most cancers [11, 13]. Moreover, TAMs have a bidirectional relationship with tumor cells and have various effects on all aspects of tumor progression, like metastasis support, tumor cell proliferation, neoangiogenesis, immunosuppression, and treatment resistance. TAMs can be classified more specifically into seven subsets: immune regulatory (Reg-TAMs), interferon-primed (IFN-TAMs), inflammatory cytokine-enriched (Inflam-TAMs), pro-angiogenic (Angio-TAMs), lipid-associated (LA-TAMs), proliferating (Prolif-TAMs), and resident tissue macrophages-like (RTM-TAMs) [28]. Each subset of the TAMs has a different duty in the TME; for instance, the Inflam-TAMs express inflammatory cytokines, such as IL1B, CXCL1/2/3/8, CCL3, and CCL3L1. Therefore, the cancer-related inflammatory response might actively recruit and regulate immune cells. As another example, the LA-TAMs can express the lipid genes profile, such as APOC1, APOE, ACP5, and FABP5, and a distinctive enrichment of lipid metabolism and oxidative phosphorylation pathways. Thus, LA-TAMs may actively inhibit anti-tumor immune responses and possibly accelerate tumor growth [29–38].

TAMs can promote cancer cell proliferation and survival by secretion of a variety of chemokines and cytokines like IL-1α, IL-1β, TNFα, TGFβ, IL-6, IL-8, CCL2, and TEXs [39–43]. IL-6 secreted by TAMs activates the JAK/STAT3 pathway, promotes tumor survival, invasion, and angiogenesis, and mediates the epithelial-mesenchymal transition (EMT) [44]. Furthermore, TGF-β, secreted from activated macrophages, has various roles in tumor progression as both pro and anti-inflammatory cytokine. For example, in the first phases, it inhibits the cell cycle and promotes apoptosis, but in the final levels, it stimulates the EMT and improves the invasion [45–48]. Also, TAM exosomes containing miR-21 impede apoptosis and promote cell proliferation in GC cells by inhibiting PDCD4 expression [42]. In these ways, TAMs have an essential role in tumor proliferation, especially in the chronic low-grade inflammatory state. Indeed, the TAMs participate in neo-angiogenesis through accumulated in the hypoxic areas after stimulated by hypoxia-inducible factor-1α (HIF-1α), producing pro-angiogenic substances like platelet-derived growth factor (PDGF), VEGF, matrix metalloproteinase (MMP), CXCL12, and angiopoietin-1. The VEGF binds to its receptor (VEGF receptor 2) and starts the proliferation and maturation of the endothelial cells (EC), improving the chemotaxis of EDs and macrophages by helping the MMP-2, MMP-7, and MMP-9 that breaking down the extracellular matrix and improve the ECs migration and create new vessel buds and progress the tumor invasion [49, 50]. Also, other factors like PDGF, CXCL12, angiopoietin-1, and TAM-derived exosomes have a variety of roles in enhancing angiogenesis in macrophage-dependent manners [51]. For example, exosomal miR-501-3p by targeting TGFBR3 assists in tube formation in pancreatic ductal adenocarcinoma (PDAC), and exosomal miR-130b-3p inhibits the expression of MLL3, therefore enhancing angiogenesis in GC cells [52].

In continue, TAMs can affect the attended cells in the TME and remodel them to a tumor-pleasant state. Indeed, they can control the expression of chemokines and promote cancer progression by employing Treg, MDSCs, and tumor-associated neutrophils (TANs) [53]. TAMs affect the immune cells by activating the Treg in the TME upon the secretion of IL-10 and TGF-β and influence the migration of Tregs into TME by releasing several chemokines like CCL5, 20, and 22. In addition, the TAMs induce the T-helper to convert the Tregs so they can inhibit the effector T cells. Also, TAMs secrete the ARG-1 and inhibit the expression of TCR complex, proliferation, memory, and anti-tumor responses in the T cells [54]. Indeed, TAMs inhibit the anti-tumor function of CD8^+^ T cells by secreting immunosuppressive factors and trigger apoptosis in T cells by expressing the ligand for the FAS death receptors. Tumor necrosis factor-related apoptosis-inducing ligand (TRAIL) [55]. Zhou et al. demonstrate TAM-derived exosomes that contain miR-29a-3p and miR-21-5p have a synergism effect in initiating STAT3 cascade signal in CD4^+^T cells and induce the Treg/Th17 imbalance, so they improve the progression and metastasis [56]. TAMs also can inhibit the NK cells by inhibiting NK cell activating receptors, regulating CX3CR1, increasing ILT-2 expression, decreasing IFN-γ and TNF-α secretion, and diminishing the migration to lymph nodes through the expression of HLA-E [57]. About the DCs, IL-10 secreted by TAMs through various mechanisms suppresses DC function, such as inhibition of their antigen presentation capacity, maturation, IL-12 secretion, inducing the tolerance and altering immunogenic into tolerogenic DCs, expressing PD-L1 and production of the VEGF [55]. Indeed, TAMs, through secretion of TGF-β, IL-6, IL-8, and IL-1β, may have a possible role in N2 (pro-tumoral) polarization recruited neutrophils in TME [58].

Indeed, TAM plays a part in cancer treatment resistance through several mechanisms, such as EMT participation, tumor angiogenesis, inhibiting T cell functions, and secretion of cytokines and chemokines [59]. In addition, TAM-derived exosomal noncoding RNAs (ncRNAs) increase resistance to antitumor drugs; for example, miR-21 through activating PTEN/PI3K/AKT signaling pathway inhibits apoptosis and increases resistance to Cisplatin in GC [60]. Also, a hypoxic condition in TME promotes exosomal miR-223 production, which is active above the pathway and enhances drug resistance in epithelial ovarian cancer (EOC) cells [61]. Also, the tumor metastasis and invasion process has several steps, such as acquiring the invasion ability, neovascularization, survival in circulation, and invasion of the different tissues [62]. The TAM has various effects at every step and improves the metastatic process. The M2-like macrophages, by their own TLR4, can produce the IL-10 that, by cooperation with IL-1β, IL-6, TGF-β, and TNF-α, increase the EMT reprogramming [27]. This change in the EMT destroyed the cell–cell junction between epithelial cells and facilitated the tumor cells into the vascular [63]. Indeed, TAMs, by expression of MMP9 and cathepsins, can break the extracellular sheet around the endothelium cells and help the invasion [64]. TAMs improve the interaction between α4-integrin and vascular cell adhesion molecule-1 (VCAM-1) on the tumor cells, so they can activate the PI3K/Akt pathway and protect the tumor cells against pro-apoptotic activities such as TRAIL [65]. Also, the tumor cells affect the macrophage cells by releasing the CSF-1 and increasing the macrophage recruitment and EGF secretion that facilitate the tumor cell's chemotaxis into the blood vessels [64, 66]. Tumor cells attract the macrophage cells in metastatic sites by releasing VEGF, CSF-1, TGF-β, and TNF-α, and these macrophages increase the tumor invasion by remodeling collagen fibers [67]. Also, the direct interaction between macrophages and tumor cells before entering them into vessels improves the extravasation mechanism [68]. Furthermore, it was shown macrophages may play a part in pre-metastatic niche formation before cancer cells arrive [69].

**Fig. 1:**
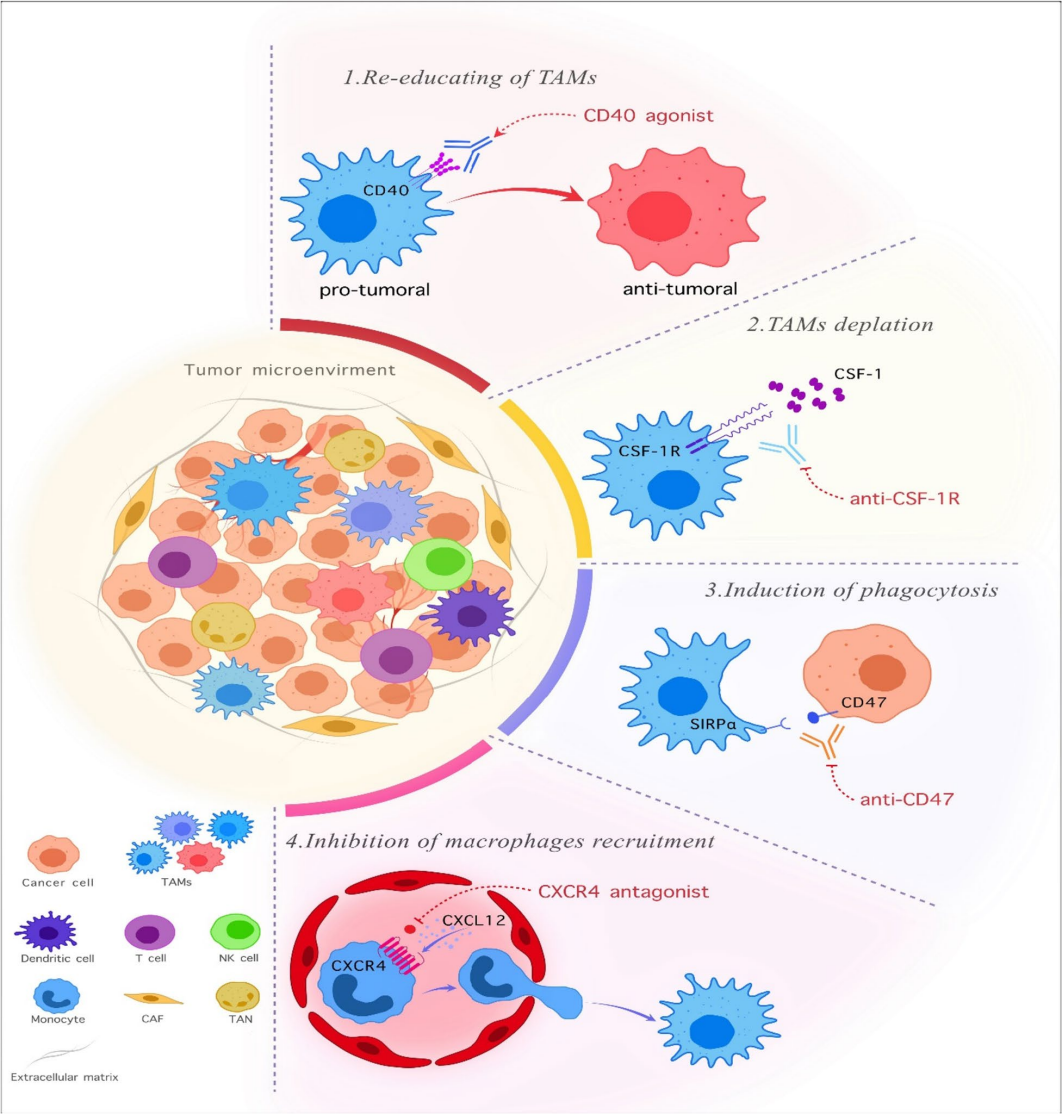
The mechanisms for modification of TAMs in the tumor microenvironment. Overall, four solutions exist for TAM-targeting to create a better anti-cancer response. As seen in the figure, the various phenotypes of TAMs have a role in the tumor progression or suppressor. Now, the two approaches have been in the target TAMs. First is the decrease in the number of TAMs in the TME by various methods like depletion of TAMs or inhibition of macrophage recruitment. Second, reprogramming the macrophages against tumor cells and repolarizing them forward the M1 phenotype and induce phagocytic activity against cancer cells. However, it requires attention. These methods are sometimes used as a combination therapy with other clinical approaches, and monotherapy has a low response in cancer treatments. TAM: tumor-associated macrophage. NK cell: Natural killer cell. CAF: cancer-associated fibroblast. TAN: tumor-associated neutrophil. CXCR4: C-X-C motif chemokine receptor 4. CXCL12: C-X-C motif chemokine ligand 12. CSF-1R: colony-stimulating factor 1 receptor. SIRPα: signaling-regulatory alpha-protein

Since the TAMs have pivotal role in cancer progression, TAM-targeting is a promising anti-tumor therapy method (Fig. 1) [70]. The TAM-targeting strategy consists of various techniques categorized based on their functions. It consists of different compartments that target multiple aspects of macrophage activity to increase the efficacy of anti-tumor therapy.

The first method is the re-education and repolarization of TAMs versus cancer cells by using some substances or receptors to activate the other part of the immune system by inducing the macrophage cells [71]. The TLRs, CD40, macrophage receptors with collagenous structure (MARCO), zoledronic acid (ZA), and PI3K are examples of this method [72–75].Recently, several therapeutics targeting CD40 are in phase I and II of clinical trials and are used widely. Also, APX005M has been FDA-approved to treat pancreatic cancer, gastroesophageal, and esophageal junction cancer with orphan drug status [76]. Another approach for the re-education of TAMs is genetic modifications like insertion of CAR-gene and IL genes to induce anti-tumoral state. For instance, the researcher transduced the IL-12 gene into macrophage structure with lentiviral approaches and showed these macrophages could experiment with better infiltration and reduce the tumor sizes and IFNγ production [77].

The second way of solution is the depletion of TAMs by target cytokine/chemokine ligand-receptor interactions [71]. An example of this is using CSF-1 receptor targeting. CSF-1R has two ligands, IL-34 and CSF-1, that have a role in the differentiation and survival of macrophage cells [78]. Pexidartinib is a CSF-1R inhibitor with FDA approval for treating symptomatic tenosynovial giant cell tumors [79]. The other drugs are Cabiralizumab, ARRY-382, DCC-3014, and TD-92 [49, 80].

The third approach for TAM targeting is the induction of phagocytes in macrophage cells [71]. The cancer cells expressing pro-and anti-phagocytic signals can control the macrophage phagocytic function. The most famous example of this approach is utilizing CD47-SIRPα interaction as an anti-phagocytic signal [81]. The interaction between CD47 and SIRP-α, called self-labeling, prevents the cancer cell's engulfment by macrophage cells and includes the ‘don’t it me’ signals [82]. The CD47 overexpressed in many solid and hematological malignancies and directly relates to poor prognosis and survival of cancer patients [83]. The studies showed that CD47 antagonists improved the phagocytic function of macrophages and antigen presentation [84]. Magrolimub, as the first drug by CD47-SIRPα interaction, blocking has distinguished results in phase I/II studies on solid and hematological malignancies and entered into phase III of studies for the treatment myelodysplastic syndrome (NCT04313881) [85]. In addition, many therapeutic approaches progressed in the CD47-SIRPα blocking, like bispecific antibodies and molecules, SIRPα fusion Mab, and small molecule inhibitors [86].

The last method is targeting chemokine axes like CCL2/CCR2, CCL5/CCR5, and CXCL12/CXCR4 [87–89]. Each of these chemokines has roles in the TAMs proliferation and cancer development, so targeting everyone with different mAbs can have anti-cancer results. For example, the CCL2/CCR2 axis has a vital role in the metastatic facilitating of TAMs, and an increased level of CCL2 is associated with many cancer types [90]. Also, CCL2/CCR2 assists in the tumor-promoting inflammation and recruitment of the TAMs in the tumor sites [91]. To target CCL2/CCR2 axis, various antagonists have been studied like CNTO888 (Carlumab, CCL2- a neutralizing antibody), MLN1202 (pozalizumab, anti-CCR2 antibody), and CCX872-B (CCR2 antagonist) [49]. Administrating the antagonist of CCL2 has a reduction effect on tumor growth, decreasing macrophage infiltration and inhibiting angiogenesis [92]. Although about all discussed methods, TAM-targeting treatment has not achieved the maximum results due to problems like heterogeneity of macrophages in TME, systemic toxicities, and difficulties in TME. So, TAM-targeted therapy sometimes does not have good results in monotherapy, and the best results were shown in combination therapies with other immunotherapy methods or routine cancer therapies [93]. So, TAM-targeted treatment was introduced as a complementary strategy in combination with other methods. The TAM targeting can enhance the chemotherapy anti-tumor function, and this combination may prevent tumor resistance to chemotherapy agents. Some previous studies demonstrated positive anti-tumor results in the TAM-targeting and chemotherapy combination. For instance, combining Gemcitabine with CSF1R inhibitor enhanced the therapy outcome and reduced metastasis [94]. Also, it showed that a combination of TAM depletion and chemotherapy can decrease tumor-vessel density properly with enhanced blood flow and chemotherapeutic agent delivery [95]. Combining Paclitaxel with CSF1R-signalling antagonists limited tumor development and metastasis [96, 97]. Also, the Paclitaxel infusion with proprotein convertase 1/3 inhibition can skew the macrophage to an anti-tumoral state, inhibit glioma proliferation, and inhibit the STAT3 pathway [98].

Indeed, combining TAM-targeting therapy with other immunotherapy methods had promising results in the various tumor surveys, like checkpoint inhibitors, nanotechnology, and ACT [99]. For instance, combining TAM-targeting and immune checkpoint inhibitors can enhance T cell function in the TME with reduced suppressive molecules, block tumor expansion, reduce immune escape, and decrease metastasis rate [54]. In a study, combining anti-PD1 with Pexidartinib could inhibit tumor expansion and CD8 + T cell actions [100]. Indeed, combining nanomaterials with TAM-targeted therapy improves their specificity and function with enhanced drug delivery, improves anti-cancer function, and skew polarizing into anti-tumoral state. For example, a layered double hydroxide nanoparticle containing miR155 could alter the M2-like TAMs and skew them to the M1 phenotype [101]. So, the TAM-targeted therapy can be promising in the combinate therapy, but with additional genetic-manipulations and strengthening, it may achieve better results in this manner.

### CAR macrophage in cancer therapy, a novel genetically modified cell in cancer treatment

The CAR M cells were introduced as a high potential cell for cancer therapy, especially in solid cancer fields, due to the unique features of the macrophages and the good abilities that CAR structure can give them. The CAR M production process and activity consist of various steps with special features, so investigating and identifying these levels helps to understand CAR M cell therapy.

### CAR structure, generations, and types for cell therapy

CAR structure consists of four major components: an antigen-binding domain (ABD), a hinge region/spacer, a transmembrane domain, and one or more intracellular signaling domains (endodomains). Each part has a particular role in the CAR function, and modification of these parts can achieve a desirable structure. The ABD part is routinely used from a single-chain variable fragment (ScFv). Still, the novel construction has utilized it from other domains such as nanobodies, designed ankyrin repeat proteins (DARPins), ligands, or receptors [102]. The ScFv is preferentially derived from murine or human monoclonal antibodies (mAbs) and composed of variable heavy (VH) and light (VL) chains connected by a flexible linker. The affinity of the ABD is another feature with different results on the antitumor efficacy of CAR structure [103]. The hinge region is an extracellular structure that links the ABD to the transmembrane, and composition or differences in the length can affect CAR-antigen binding and signaling. The transmembrane domain docks the CAR in the cell membrane and can affect CAR expression, stability, and function [104]. Lastly, the intracellular signaling domain receives upstream signals from the transmembrane domain and triggers downstream signaling pathways that finally lead to the anti-tumor function of the modified cell. The CAR structure between macrophage and T cells is the same but differs in the intracellular domain. For example, CD147, FcRγ, and Megf10 have been investigated more specifically in CAR-Ms, but the CD3ζ is a common intracellular domain utilized in both [105, 106].

According to the architecture of the intracellular domain and secretion profile, the CAR structure has been designed for five generations. However, the first three are used in CAR-NK and CAR-M production. The first generation has a single intracellular domain that usually consists of the CD3ζ signaling domain but has limited efficacy and insufficient activation signal. Therefore, the second and third generations added one or two co-stimulatory domains (CD28, 4-1BB, CD27, or OX-40), respectively [107]. Fourth-generation CARs, T cells redirected for universal cytokine-mediated killing (TRUCKs), were designed based on the second-generation CARs with the ability to induce secretion of transgenic cytokine (IL-7, 12, 15, and 18) through the nuclear factor of activated T-cells (NFAT), that causes a proper immune response by inducing the various anti-tumor mechanisms. In the fifth generation, the structure can release factors like ILs for better function. This goal has been achieved by localized cytokine signaling only in the existence of the target antigen. The construction of fifth-generation CAR-T was also based on the second-generation CARs with the addition of an IL-2Rβ fragment that can initiate Janus kinase/signal transducer and activator of transcription (JAK-STAT3/5) pathways. These pathways promote CAR persistence, proliferation, activation, and localized cytokine signaling [2, 108]. As transmembrane domains, the studies usually used CD8α, CD147, and CD28 and for intracellular domain utilized from CD3ζ, Megf10, OX40, CD28, 4-1BB, CD86, CD147, TIR, toll like receptor (TLR), Bai1, PI3K, MerTK, MYD88, and FcRγ [109]. Each of these domains gives special properties to CAR M. For instance, the FcRγ receptors can mediate antibody-dependent cellular phagocytosis (ADCP) by the connection between the Fc segment and IgG antibody [110, 111]. Moreover, to generation, the CAR has been categorized based on the function into various types such as multi-specific CARs, TCR-CARs, converter CARs, universal CARs, and inducible CARs [112].

### Manufacturing and gene-delivery of CAR M

**Fig. 2:**
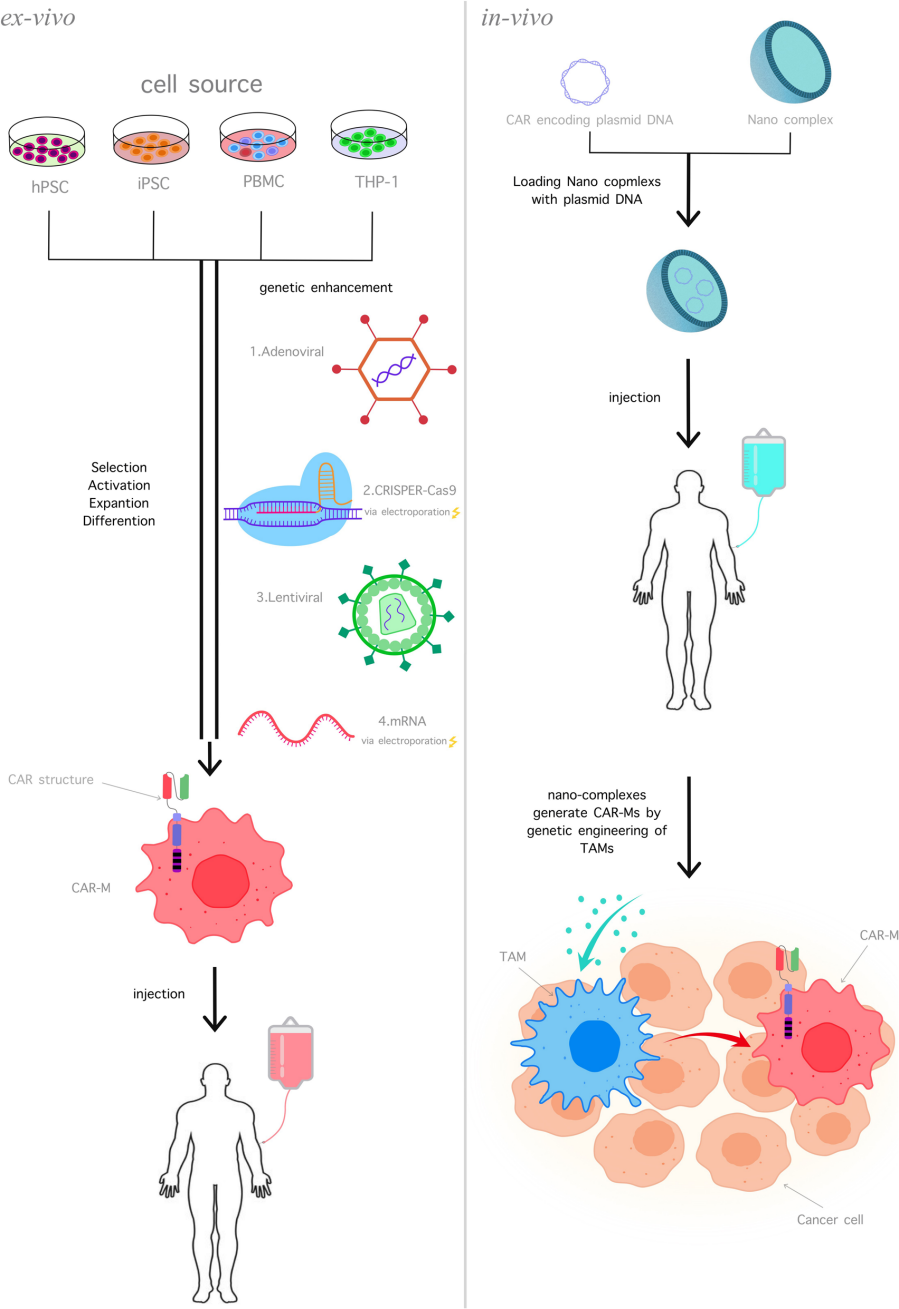
The manufacturing of CAR M cells. The CAR M cell production can be done in two separate manners, the in-vivo and ex-vivo manners. In the ex-vivo way (left), the cells were chosen from the different cell sources and then modified with genetic-engineering tools for CAR M manufacturing. This process requires cell selection, activation, expansion, and differentiation. Then, the CAR M can be administrated by intratumoral or intravenously. In the in-vivo manner (right), the CAR transgene is induced into nano complexes, and then the final structure can be injected into the body for transforming the TAMs to CAR M cells in the TME for maximum effect

The manufacturing process of CAR-M cells is like that of CAR-T and CAR-NK cells. First, the cells were collected from a promising source, and with unique gene-modified methods, the CAR-gene was inserted into the cell, and the achieved cells were transformed to the culture environment for cell expansion; then the CAR-M cells were ready to administrate into the body (Fig. 2).

Macrophage cells have high resistance to gene engineering due to their high capability in foreign nucleic acid detection. However, the development in genetic manipulation makes it possible to apply viral and non-viral methods [113]. Early, HIV-1-based lentiviral vectors failed to infect myeloid lineage cells due to SAMHD1, a myeloid-specific HIV-1 restriction factor that decreases the deoxynucleotide pool and hinders reverse transcription. To solve this problem, viral accessory protein Vpx has been used that binds to SAMHD1 and induces its degradation [114, 115]. In brief, some preclinical studies utilized lentiviral vectors in CAR-Ms manufacturing; for instance, Lei et al. designed iPSCs-derived CAR-Ms (CAR-iMACs) by transducing CAR into induced human pluripotent stem cells (ihPSCs) or researchers developed CCR7-targeted CAR M by lentiviral transducing. Adenovirus is another vehicle for macrophages that have been utilized as viral vectors. The high expression of CD46 in macrophage surfaces mediates the docking protein for group B adenoviruses, such as Ad35 [116, 117]. Next, a replication-incompetent chimeric adenoviral vector Ad5f35 was evaluated as gene-delivery to macrophages that expressed CAR with high efficiency [105]. Ad5f35 triggered the activation of the macrophage inflammasome and conferred a favorable proinflammatory priming stimulus that has a synergic effect with CAR activity with M1 phenotype stabilization [118]. Klichinsky et al. transduced CAR-gene efficiently into macrophages by adenoviral vector Ad5f35, generating CAR-M with sustainable M1 phenotype. However, clinical uses of viral vectors are highly restricted because of concerns for oncogenic alteration and immune reactions [119, 120].

In other approaches, the CAR gene transferring can be done with non-viral methods like electroporation and nano-complexes that generate CAR M into hPSCs or macrophages. For example, knocking the anti-GD2 CAR gene into hPSCs with electroporation of CRISPR/cas9 created the CAR M versus the GD2^+^ cancer models [121, 122]. Also, some studies use nanocarriers to transfer the plasmid CAR and integrate that into the DNA via a transposon system to generate CAR M *in-vivo* [123–125]. In addition, Ye et al. designed LNPs that contain CAR mRNA and generate anti-CD19 CAR M by transferring LNPs to murine primary macrophages, demonstrating notable cytotoxic effects against human B-cell lymphoma *in-vitro* [126]*.* In another study, researchers delivered mRNA via electroporation into macrophages, designed anti-HER2 CAR Ms, and utilized IFN-β, resulting in a sustainable M1 state, promoting CAR expression, and boosting anti-tumor activity [127].

Various factors affect the CAR M polarization state during the manufacturing process [105, 128, 129]. For instance, cytokines applied during the CAR M manufacturing process, like GM-CSF, M-CSF, and IL-1β, are linked to the M1 state differentiation [121, 130]. Also, the type of vector utilized for the transduction can affect macrophages' polarization state; for example, researchers used the Ad5f35 vector, which can induce an M1 polarization state in macrophages independent of CAR transgene [105]. Indeed, the CAR structure interferes with the macrophage state and skews them into M1 polarization states with different anti-tumor potency among various CAR structures [106, 128, 131]. The cell source is another factor in the macrophage state; for example, surveys showed the CAR M cells derived from iPSCs tend toward the M2 state in the target antigen absence; however, in the presence of the target antigen, they shift toward the M1 phenotype [129]. Although studies showed in the presence of antigen-bearing tumor cells, CAR M cells exhibited the anti-tumor effect independent of the M1/M2 state [121, 129].

### Various sources and functions of CAR M cells

One of the crucial factors in cell therapy is the source of cells. Some cells, like T cells, are limited in the autologous cell source due to the alloreactivity and possibility of creating GVHD. Indeed, the collected cells from cancer patients, due to the tumor cell secretions and previous heavy treatments, are fragile and do not have enough anti-tumor functions. Thus, in the autologous cell collected manner, personalized cell sources create a high cost of treatment and reduce the “off-the-shelf” potential [132]. The NK and NKT cells have overcome in this field because they have several sources like autologous, homogenous, peripheral blood mononuclear cells (PBMC), umbilical cord blood (UCB), induced/human pluripotent stem cells (iPSCs/hPSCs), and cell lines [133].

Macrophages have various sources, like NK cells, but the studies about the different cell fountains are few. The first macrophage cell source is the PBMCs, an easy-access source enriches with various immune cells for immunotherapy. These cells can produce many pro-inflammatory factors like IL-6, IL-8, and TNFα, so they can exhibit more surface markers like natriuretic peptide receptor (NPR), CD14, and CD68 that give more inflammatory function than cell lines. However, the PBMCs have a low genetic manipulation rate and, in the clinic, have less feasibility [134]. Cell lines like THP-1, a human leukemia monocyte cell line that shows the morphology features and functional abilities of macrophages, can be utilized as cell sources. This cell line converts to macrophage-like cells by inducing the secretion of substances like phorbol-12-myristate-13-acetate (PMA), 25-dihydroxy vitamin D3, and macrophage colony-stimulating factor (M-CSF). THP-1 can express the typical myeloid cell surface markers by inducing LPS and IFN-γ and transforming to the M1 phenotype. Indeed, the THP-1 cell line has a homogenous genetic background, so it is easier to culture, has rapid proliferation, and has higher safety [135]. Although the THP-1 is similar in the genetic construction to PBMC-derived macrophages, they did not have the same features. For instance, the THP-1 does not have LPS tolerance due to the upregulation of some of the NF-ĸB gene carriers.

Furthermore, THP-1 does not secrete the IL-6 and IL-10 and secretes low IL-8 under polarization to M1, so they are a better option than PBMCs [136, 137]. Another source for CAR M is primary human hematopoietic stem and progenitor cells (hPSCs), a logical substitute for large-scale CAR M production and improvement of “off-the-shelf” immunotherapy. The macrophage cell collected from hPSCs is functional, with uptake yeast particle ability and high plasticity features to convert M1 or M2 phenotypes. Indeed, the hPSCs-derived macrophage cells can unlimitedly expand in the culture and produce numerous cells by one established hPSC line. Furthermore, the hPSCs can be subjected to multiplex editing approaches for introducing multiple genetic traits and colonies selected for the homogeneity of gene editing [138]. The last cell source is iPSCs collected from patients' cells and differentiated into various cell types. This macrophage source may create large-scale CAR M cells. The differentiation of iPSC macrophage requires some protocols that Lyadova et al. reviewed in their work [139]. The standard method for differentiating iPSC-derived macrophages is using the embryoid body formation with specific modifications that induced the myeloid macrophage by cytokines. Also, the HSPCs used for differentiation to iPSC-macrophage, but for this act required more large cytokines. So, the scientists devised new protocols using a feeder-free suspension system to solve this problem [140]. The immune cells derived from iPSCs are good sources due to their high flexibility in expansion and genome editing [141]. So, the first step in the iPSC-derived CAR M (CAR-iMAC) cells has been done by reprogramming PBMCs back to iPSC with non-integrative episomal vectors and lentiviral ones for CAR-gene inducing. These cells could exhibit significant anti-tumor efficacy against the tumor cell lines [142]. The advantages and disadvantages of all sources are mentioned in Table 1. Although the macrophages have various sources for producing CAR-Ms, more study is needed for knowledge about every source.

**Table 1:** A different source of macrophage to CAR M production. The macrophage cells have various sources for utilization in CAR M production, so this can improve their off-the-shelf potential

*Source*	Properties	Benefits	Disadvantages	References
*Macrophage cell line*	THP-1 cell line	1. Have a homogenous genetic background 2. Not seen LPS-induced tolerance 3. Lower secretion of IL-8 and without production of IL-6 and 10	1. Cannot stay in the previous morphology 2. Low feasibility in the clinic	[135]
*Human macrophages from PBMCs*	Peripheral blood was collected, and macrophage cells were isolated by apheresis	1. Can stay in the previous morphology 2. More inflammatory properties due to expression surface markers 3. potent anti-tumor efficacy	1. Seen LPS-induced tolerance 2. Low potential for cell- engineering 3. High heterogeneity after gene-editing 4. Limited cell resources for particular malignancies 5. Donor dependent 6. High risk for GVHD outbreak	[135, 142]
*HPSCs*	The hPSCs can be collected from bone marrow, cord blood, peripheral blood after G-CSF mobilization, and an extensive bank of HLA-typed donors	1. High polarization ability to convert M1 and M2 phenotypes 2. Unlimited expansion in the cultural environment 3. Subjected to multiplex editing approach	1. In peripheral blood collection, it can affect previous treatments 2. Complicated manufacturing process	[121, 143]
*iPSCs*	Collecting the patient's blood cells and differentiating into various cell types	1. High polarization ability in M1 cell phenotypes 2. High potential of cytokine secretion in an antigen-dependent manner 3. High phagocytosis function 4. High flexibility in the expansion and genome editing	1. Production of some pro-inflammatory cytokines like IL-1β, IL-6, and IL-12 and possibility the creation adverse effect 2. Complicated manufacturing process	[142, 144]

*Abbreviations*:* THP-1* Human myeloid leukemia mononuclear cells, *PBMCs* Peripheral blood mononuclear cells, *HPSCs* Primary human hematopoietic stem and progenitor cell, *iPSCs* Induced pluripotent stem cell, *G-CSF* Granulocyte colony-stimulating factor, *LPS* Lipopolysaccharide

**Fig. 3:**
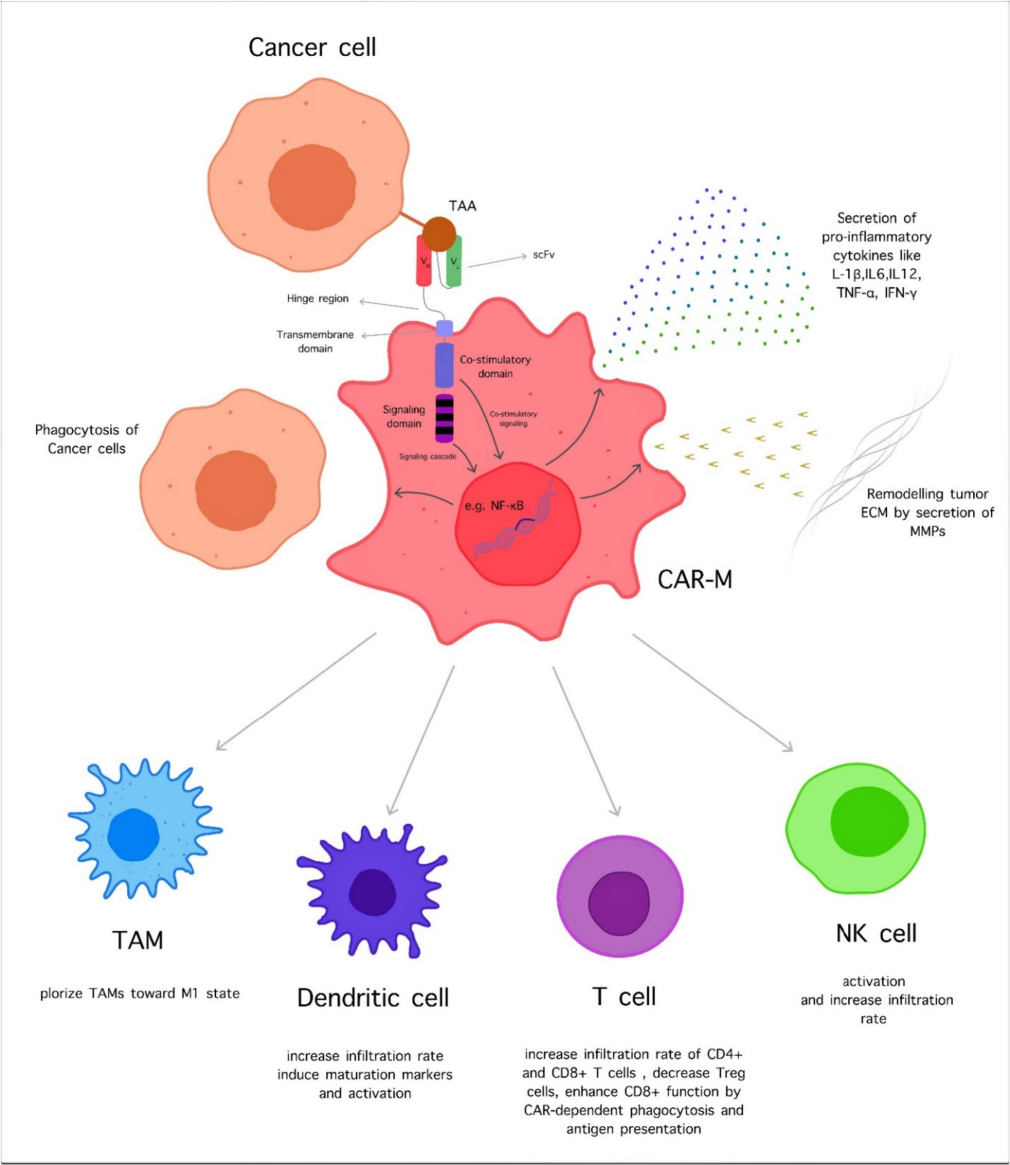
The CAR M cell functions. Above: The CAR M cells can recognize the tumor cells with TAA-CAR interaction and have various reactions such as secretion of different pro-inflammatory cytokines, induction phagocytosis, and help to remodel tumor ECM by MMPs. Below: The CAR M cells, after activation, can promote the other compartments of the immune system like TAMs, DCs, Ts, and NK cells by different methods to increase the anti-tumor function

The CAR-M cells and their killing ability can modulate and regulate the immune system and related factors to improve their anti-cancer property (Fig. 3). CAR-M changes the TME factors, inducing the proinflammatory signals by upregulating MHC genes and TNF expression. Also, the CAR-M can convert the M2 macrophages to the M1 phenotype, while the M2 macrophages cannot induce the CAR-M toward the M2 phenotype. This effect is crucial in treating solid tumors due to the high number of TAMs. Furthermore, the M2 macrophages cannot affect the anti-cancer activity of CAR-M, so the immunosuppressive compartment of TME has failed [105]. The CAR-M cells can interact with the immune cells and can stimulate them. For example, by the antigen-presenting ability, CAR-M stimulates the T cells compared to the natural macrophages so that they can elevate the cytotoxicity rate. The interesting point is the ability to activate T cells by CAR M is not a limitation to activated T cells and involves the resting T cells [105, 145]. Indeed, recognition of tumor-associated antigen (TAA) with CAR structure induces the phagocytotic action versus cancer cells, increasing the tumor-killing capacity. Also, the application of CAR-M cell therapy is not limited to malignancies, and several studies utilize CAR-M cell therapy for other diseases such as Alzheimer, SARS-COVID virus-2 (SARS-CoV-2), and Periprosthetic joint infection [146–148].

### The race between CAR macrophage cells and other CAR-armored cells

Each one of the CAR-armored cells has various features and different advantages and disadvantages. By the progression in the cell gene modifications, these remarkable properties assign that one is used based on the cancer type, patient situation, tumor grading, and healthcare state. The three famous cells discussed here are CAR T, CAR NK, and CAR M. However, the other cells, such as CAR NKT and CAR neutrophil, introduced the basis of cancer immunotherapy and have significant results in the *in-vitro* and *in-vivo* studies (the CAR NKT comparison done in Table 2) [149, 150]. In the first look and hypothesis, the innate member immune system cells seem to be the better option for CAR-base immunotherapy and have more flexibility in function and production. Still, the CAR macrophage looks more like CAR T than other CAR-innate cells. But, the data requires more studies for the last decision [145]. There are different factors for comparing the CAR-armored cells to answer this question, which is suitable for various situations (Table 2).

**Table 2:** The comparison between the different famous types of CAR-armored immune cells. Cells like NK, NKT, and macrophage could be another alternative for T cells in a CAR-structure anti-cancer manner

	**CAR-T**	**CAR-NK**	**CAR-NKT**	**CAR-Macrophage**
**Intracellular signaling domain**	CD3ζ + Costimulatory domains (CD28,4-1BB, CD137)	Like CAR T cells + NK-specific signaling domains (DAP10, DAP12, CD28,4-1BB,2B4)	Like CAR T cells + NK-receptors signaling domains + TCR-receptors signaling domains	Like CAR T cell + ITAMs associated signaling domain + Other ligands for modifying TME
**Transmembrane domain**	CD8, CD28	NKG2D, CD8, CD28	CD8, CD28	CD28, CD147, CD8
**Source**	Autologous or MHC-I matched allogeneic	Autologous / NK cell lines/ non-MHC-I allogeneic/PBMCs/UCB/ hPSCs/iPSCs/BM	PBMCs UCB hPSC/iPSCs	Autologous/ iPSCs/ hPSCs/Cell lines/BM/UCB/THP-1 cell line
***In-vitro* expansion**	Yes	In autologous: ok Cell lines: needed expansion before transduction	In autologous: ok Cell lines: needed expansion with α-GalCer-pulsed	In autologous: ok In iPSC and cell lines: required to expand before transduce
**Transduction efficacy**	Higher	Low	Low	Low
**Cytokines are used for cell expansion**	IL-2	IL-15	IL-2	GM-CSF
***In-vivo* controlling of proliferation and expansion (utilizing suicide genes)**	Needed	Easier or not needed	Not be required commonly	Probably needed
**Repeat activation upon first antigen exposure**	Slow	Fast	Fast	Fast
**Life span and persistence**	High life span and long-term persistence	Low life span and limited persistency	Low life span and long-term persistency	Increased life span with limited persistency in circulation
**Repeat doses**	Not needed	Needed	Needed	Not needed
**Tumor infiltration**	Usually, poor	Usually, poor	Usually, poor	Very abundant
**Cytotoxicity effect**	High	Low	High	High
**Cytotoxicity manner**	CAR-dependent	Both CAR-dependent and CAR- independent	Cytotoxicity was dependent and independent of CAR The indirect anti-tumor function	Both CAR-dependent and mediated alteration of TME and mediate immunostimulatory TME and APC stimulate the immune response
**Cost**	High	Low	Low	Low
**Off-the-shelf**	Not significantly	Significant with cell lines With autologous cells possible while poor recovery or cryopreserve	Potential off-the-shelf CAR-cell products	Possible with a different source of macrophage
**Clinical outcomes**	Proven with six FDA-approved drugs	Proven but without any approval therapy or drugs	Unproven but based on the clinical results, guess with significant action	Unproven but based on preclinical results guess with effective action
**Efficacy in solid tumors**	low	Moderate	Moderate	High
**Side effect**	Common and often with the fatality	Less common and usuriously risk	Less common and ungraciously risk	It is expected to be shared without clinical evidence with low fatality potential

*Abbreviations*:* NK* Natural killer, *DAP* DNAX activating protein, *TCR* T cell receptor, *ITAM* Immunoreceptor tyrosine-based activation motif, *TME* Tumor microenvironment, *NKG2D* Natural killer group 2 member D, *MHC* Major histocompatibility complex, *PBMC* Peripheral blood mononuclear cells, *UCB* Umbilical cord blood, *hPSC* Human pluripotent stem cells, *iPSC* Induced pluripotent stem cells, *BM* Bone marrow, *IL* Interleukin, *GM-CSF* Granulocyte–macrophage colony-stimulating factor, *CAR* Chimeric antigen receptor, *APC* Antigen-presenting cell, *FDA* Food and Drug Administration

Some limitations are common among all CAR-armored cells; approximately any have unique benefits. These barriers are sometimes related to the innate features of the tumor cells and are more predominant in solid cancer cells, like lack of special tumor target receptors, physical barriers, tumor heterogeneity, and antigen escape. Examples of these problems can include the “on target-off-tumor” side effect (means of recognition of normal cells in the body via the same targeted antigen on the CAR structure) or tumor heterogenicity. Although targeting some antigens in solid cancer, like melanoma antigen recognized by T cell 1 (MART1) and glycoprotein 100 (Gp100) by CAR-T cells, could have beneficial anti-tumor results, however due to the occurred “on target-off-tumor” side effect, these studies had weak anti-tumor effects and not required results [151, 152].

The advantages of the CAR-M cells begin from the TME-related anti-immune cell factors. Despite the CAR-M cells, the CAR NK and CAR T cell, under the effect hostile of TME, lose their functions. The inhibitory actions of the existing cells like TAMs, Treg, MDSCs, and cancer-associated fibroblasts (CAFs) occur by producing various cytokines and creating an immune suppressive environment [153]. For example, the VEGF, besides neovascularization, can have roles in all aspects of the repressive immune system [154]. The CAR M does not undergo exhaustion and has a better anti-tumor function in TME [123, 131].

Furthermore, the high macrophage phenotype plasticity rate allows them to transform into different phenotypes based on environmental stimulators [155]. The other comparison factor is the ability to traffic and infiltrate into tumor sites. The abnormal vascularization with improper adhesion molecule expression suppressed CAR T cells' adherence, migration, and infiltration into tumor sites. Also, the dense external matrix that included CAFs as a physical barrier and inhibitory cytokines in TME can reduce the infiltration of CAR T cells [156]. Indeed, solid tumors are mostly cold tumors, meaning the infiltration rate and attended cells into TME are deficient [157]. These problems exist in CAR NK therapy, too, though the cooperation by some ILs like IL-15 can promote the CAR NK cell infiltration rate into TME [158]. In contrast, the CAR-M cells have better action in trafficking and infiltration and can handle TME immunosuppression better, so they exhibit distinguished function in TME [153, 159].

The expansion and cell persistence are the other essential factors in CAR manufacturing and differ among the cells. In addition, survival *in-vivo* is crucial, especially in cancers requiring a lengthy treatment process like ALL [160]. The lack of CAR T cell persistence and expansion is a vital problem and barrier in CAR T cell manufacturing and functions. This phenomenon may be related to the host anti-transgene immune response to CAR T cells and can be improved by using drugs like Fludarabine [161]. Indeed, the *ex-vivo* culture could promote the CAR T cell expansion but did not help after administrating into the body, and cells undergo exhaustion and are susceptible to the activation-induced cell death (AICD) process after repeat antigen stimulation [162, 163]. Also, CAR NK cells have low persistence in the body, and the average life span of the CAR NK cell (average is two weeks) is lower than CAR T and CAR M, so this acts as a double-edged sword in CAR-dependent therapy because it reduces the on target-off tumor. In contrast, it can have less effect in some cancers with long treatment periods and require repeated doses of the cells [164].

The outbreak of side effects is the other deciding factor in the treatment choice. Although the high response rate of CAR T cells in the refractory and resistance tumors, this response will not be without complications. Systemic cytokine toxicity occurs after CAR T cell administration in the acute phase of the cytokine release as a physiological cell response and creates CRS toxicity [165]. Furthermore, ICANS can occur due to the elevation of cerebrospinal fluid cytokine levels and disruption in the blood–brain barrier [166]. In addition, CAR T cells can induce hemophagocytic lymphocytosis (HLH)/Macrophage activation syndrome (MAS) toxicity. Based on previous studies, HLH/MAS can occur in 3.5 percent of patients, managed by corticosteroid-base treatment is insufficient, and the fatality is high [167].

In contrast, the CAR T and CAR NK cells release the cytokines by different cell signaling and different cytokine production profiles; for example, the CAR T cell, after activation, releases inflammatory cytokines like TNF-α, IL-1β, IL-2, and IL-6 while the CAR NK cells did not release any of them and production other substances like GM-CSF [168]. So, the possible outbreak of CRS and neurotoxicity effect is low. In a study on 11 patients with HLA-mismatched anti-CD19 CAR NK cells, none showed any sign of CRS or neurotoxicity [168]. However, in a survey by CAR NK-92 cells versus non-small cell lung cancer (NSCLC), one of the treated patients showed the CRS manifestation, and this was approved by the additional evaluation [169], so maybe the CAR NK cells can lead to the CRS by a different method, but the demonstration of rate in lower than CAR T cells. The studies about CAR M cells did not reveal any dangerous side effects except for several weak reactions like a low-grade fever, abdominal discomfort, cutaneous toxicity, and body weight loss, so any significant toxicity side effects were not shown. However, CAR-Ms have a low risk of GVHD due to rapid extravasation from blood vessels and *in-vivo* limited expansion capacity [170]. In a preliminary result from phase I/II of a clinical trial named IMAGINE, the first results showed no sign of CRS or another cytotoxicity (NCT05138458). In addition, a study used CAR-M against SARS-COVID virus-2 (SARSCoV-2) by the MERTEK kinase as an intracellular domain *in-vivo*. These CAR-M cells could improve cell elimination without upregulating proinflammatory cytokine secretion [147]. Although the recent results have not shown the side effects of CAR, more study is needed to approve this subject.

Regarding the anti-tumor function, the CAR NK cell, in contrast to the CAR T and CAR M cells, has a double function; its mean cell can eliminate the cancer cells in a CAR-dependent and independent manner [156, 171]. In the CAR-independent way, the cells utilized the innate receptors; for example, the CAR NK cells used from the activator and inhibitory and can kill the cells or utilized from the CD16 receptor and bind with the Fc portion of IgG to initiate the ADCC (antibody-dependent cellular cytotoxicity) process against cancer cells [172]. Indeed, CAR NK and CAR M cells don’t need the MHC-matching and can be used in the “off-the-shelf” ready-to-use and personalized treatment, so treatment by this method can reduce the cost of treatment[156]. Indeed, CAR NK and CAR M can have regulatory functions on immune system activation and improve their situation. For example, the CAR M can increase the antigen-presenting, so increase the T cell cytotoxicity, or the CAR NK cells by production of the IFNγ can start a cascade in the immune system and activate the DCs and T cells [159].

The other comparison factor is the cell sources; each cell has various sources that can be more useful. In a study on CAR T cells, 22.5 percent of patients lost the chance of the treatment due to a long time of collection and manufacturing process [173]. In contrast, CAR T cells, CAR NK, and CAR-M cells have many diverse cell sources that create an excellent condition for “off-the-shelf,” speeding up cell preparation and decreasing the treatment cost [133]. The same structure is usually applied to CAR T cells in comparing the CAR structure. However, the studies exhibited that due to the discrepancy in intracellular signaling or innate features of cells, using a particular intracellular domain or memorable transmembrane domain can promote anti-cancer abilities. For instance, NK cell-specific intracellular signaling domains such as 2B4 or DAP 10 and DAP 12 or utilizing NKG2D as a transmembrane domain can boost the CAR NK cells actions [174]. The macrophage cells can use the CAR T structure, while the different signaling pathways are seen between them. For instance, the CD3ζ can activate the CAR M cells, but differently; in CAR T cells, it phosphorylated the immunoreceptor tyrosine activation motifs (ITAMs) by Src family kinase and binding to tandem SH2 domain in kinase ZAP70, while the CAR M does not express ZAP70 and another kinase Syk [175]. Indeed, in addition to CD3ζ, other ITAMs-containing intracellular domains like FcRγ and Megf10 can induce the phagocytic function in macrophages. FcRγ can transduce the canonical signaling for ADCP, and Megf10 has a role in the phagocyte apoptotic cells [105, 176].

### Pre-clinical studies of CAR Macrophage

The CAR M cells *in-vitro* and *in-vivo* evaluation demonstrated significant results in hematological and solid cancers. The macrophages utilizing CAR structure could have more robust anti-cancer features than control macrophages or other macrophage-based treatments. The studies showed promising results of cytotoxicity in the CAR-manner by various target antigens such as Disialoganglioside (GD2), epidermal growth factor receptor (EGFR) III, CD19, transmembrane glycoprotein mucin 1 (MUC1), human epidermal growth factor receptor 2 (HER2), and mesothelin.

The preclinical studies of CAR M cells demonstrated notable anti-tumor action *in-vitro* and *in-vivo* investigations (Table 3*)*. For example, CAR M showed anti-tumor effects on leukemia cells with luciferase gene expression or ovarian cancer cell line HO8910 expressing high mesothelin *in-vivo* [129]. Indeed, MUC1-targeting CAR Ms showed potent anti-tumor function by phagocytosis and secretion of pro-inflammatory cytokines such as IL-1β, IL-8, and TNFα in the presence of MUC1 expressing tumor cells from solid lung tumors or malignant pleural effusions [177]. Furthermore, CAR M phagocytes and eliminated HER2^+^ ovarian cancer cells in an antigen-dependent manner were *in-vitro*, significantly decreasing tumor burden and improving overall survival in xenograft mouse models [105]. Zhang et al. designed HER2-targeting CAR M cells with CD147 intracellular domain as a necessary factor for ECM altering. Hence, the CAR M destroys the TME physical barrier via the expression of MMPs. Engineering macrophages with CAR-147 significantly increased the expression of multiple (Table 4) MMPs like MMP3, MMP11, MMP13, and MMP14 without affecting phagocytosis and reactive oxygen species (ROS) production. CAR-147 macrophages did not prevent the development of tumor cells *in-vitro* but significantly inhibited tumor growth *in-vivo*. CAR-147 macrophages remodel the tumor ECM, break down the TME physical barrier, and promote T-cell infiltration, inhibiting tumor growth. The levels of the cytokines TNF-α and IL-6, which are inflammatory key factors in CRS, were substantially reduced in the peripheral blood in mice treated with CAR-147 macrophages; however, the levels of IFNγ and IL-12, which have the anti-tumoral function, notably increased [178].

**Table 3:** Summary of the critical findings of preclinical CAR-M studies

**Targeted- cancer or cell line**	**Genetic enhancement**	**CAR structure**	**Results**	**Ref**
**Melanoma** **Neuroblastoma**	CRISPR/cas9	hPSCs-derived GD2 targeted with CD3ζ-CD28-OX40	*In-vitro* results showed substantial anti-tumor activity The neuroblastoma tumor burden is reduced in the xenograft mouse model with minimal adverse effects	[121]
**Ovarian cancer** **Pancreatic cancer** **Leukemia** **Lymphoma**	Lentiviral	iPSCs-derived CD19-targeted CD86-FcγRI or iPSCs-derived mesothelin-targeted-4-1BB-CD3ζ	*In-vitro* CAR-iMacs showed cytokines expression and phagocytosis in an antigen-dependent manner and polarized them toward an M1 state *in-vivo* result CAR-iMacs showed some anti-tumor effect on leukemia cells expressing and high mesothelin-expressing ovarian cancer cell line	[129]
**Lung tumors** **Plural effusion malignant cells**	Lentiviral	THP-1 derived-MUC1 CAR-M CD3ζ-CD28-OX40	*In-vitro* results showed potent anti-tumor function by phagocytosis and secretion of pro-inflammatory cytokines such as IL-1β, IL-8, and TNFα	[177]
**Breast cancer**	Lentiviral	HER2-targeting-CD147 CAR-M	*in-vitro*, CAR-147 macrophages did not inhibit the growth of tumor cells *in-vivo* significantly inhibited tumor growth, remodeled the tumor ECM, and promoted T-cell infiltration, resulting in tumor growth	[178]
**Ovarian cancer**	Adenoviral vector (Ad5f35)	THP-1 derived HER2 CAR-M	*In-vitro* sustainable M1 phenotype and polarize M2 toward M1 phenotype. CAR-Ms phagocyte and eliminate tumor cells in an antigen-dependent manner Significantly decrease tumor burden and improve overall survival in xenograft mice models	[105]
**Mesothelin-positive tumor cells**	Adenoviral vector (Ad5f35)	PBMC-derived mesothelin CAR-M with CD3ζ (CT-1119)	*In-vitro*: high CAR expression, possesses M1 phenotype with relative resistance to M2 shift, and exhibits robust tumor cell-killing capability and pro-inflammatory cytokines Substantially decreased tumor burden in a murine xenograft model of lung cancer	[179]
**Neuroblastoma**	Nano complex	Anti-ALK CAR-M with- CD3ζ-CD28- IFN-γ gene	*In-vitro*: Transfection with MPEI/pCAR-IFN-γ did not very significantly polarize macrophage toward the M1 phenotype The MPEI/pCAR-IFN-γ injection into Neuro-2a tumor-bearing mice reduced tumor growth, decreased the Treg cell, and increased the function of activated CD8^+^ T cells in the tumors	[123]
**Glioma**	Nano complex	Anti-CD133 CAR-M with- CD3ζ	NP-pCAR induces M1 polarization and increases the secretion of IL-1β and TNF-α of targeted macrophages *in-vitro*. Also, it decreases the number of CD133-positive tumor cells and causes tumor regression without traceable toxicity in the orthotropic mouse glioma model	[125]
**Brain stem glioma**	Nano complex	Anti-HER2 CAR-Ms	*In-vitro*: PC_D68_/PB/N/R nanoparticles produce CAR-Ms with M1 phenotype and greater phagocytic and cytotoxic ability in an antigen-dependent manner. Intratumoral injection of P/PB/N/R nanoparticles generates Anti-HER2 CAR-Ms, causes phagocyte tumor cells, enhances immune system response, and tumor regression in mice without any notable side effects	[124]
**EGFRvIII positive cells**	Lentiviral	IPSCs derived anti-EGFRIII CAR M with different intracellular domains, including CD3ζ, TIR, and CD3ζ-TIR	CAR-iMACs demonstrate significant anti-tumor activity, but TIR-CAR-iMACs showed more pro-inflammatory activity, killing persistence, and greater anti-tumor activity *in-vivo*. In addition, the CD3ζ-TIR-CAR-iMACs could increase anti-tumor activity and contribute to retaining M1 polarization of CAR-iMACs, more robust anti-tumor capability compared to alone them *in-vivo*	[128]
**4T1 tumor cells (breast cancer)**	Lentiviral	RAW264.7-derived anti-CCR7 CAR M with domain derived from TLR2, TLR4, TLR6, MerTK or 4-1BB-CD3ζ	MerTK-CAR-M displays targeted anti-tumor function in both *in-vitro* and *in-vivo* and exhibits most phagocytic and anti-tumor activity against tumor cells by reducing tumor burden, increasing median survival time and creating the inflammatory environment by increasing the levels of proinflammatory cytokines in serum *In-vivo*	[131]
**Raji B cells**	Lentiviral	J774A.1 cell line Anti-CD19/CD22 CAR-P with different intracellular domain including Megf10, FcRγ, Bai1, and MerTK with/without PI3K	CAR-Ms with the Megf10 or FcRγ intracellular domains demonstrate the greater phagocytic capacity of CD19-positive cells compared to others PI3K CAR-P induced some whole-cell phagocytosis, comparable to the CAR-P-FcRγ. Also, CAR-P tandem macrophages notably decreased the number of tumor cells. However, the CAR-P tandem was much more efficient at whole-cell phagocytosis than the CAR-P-FcRγ	[106]
**CT26-HER2 cell line**	Adenoviral vector (Ad5f35)	BM-derived anti-HER2 CAR M cells	CAR M cells showed anti-tumor activity and phagocyte HER2-overexpressing tumor cells and boosted the cytotoxicity of CD8^+^ T cells by inducing MHC-I expression on tumor cells. *In-vivo*, CAR-M causes significant tumor regression, improves overall survival, increases infiltration rate of CD4^+^ and CD8^+^ T, NK, and DC cells in the TME, and ameliorates epitope spreading, improving T-cell reaction to TAA	[180]
**Raji cells**	Lentiviral	BM-derived anti-CD19 CAR M with different intracellular domains, including Megf10, PI3K, and FcRγ	CAR-M PI3K and CAR-M FcRγ exhibit more phagocytic capacity; CAR-M FcRγ possesses more substantial cytotoxic and phagocytic ability. Also, the utilization of CAR-M FcRγ and CAR-T cells together showed substantially greater cytotoxic power than CAR-M FcRγ or CAR-T cells alone, and inflammatory factors such as IL-1β, IL-6, IFN-γ, CXCL1, MCP-1, and MIP-2 have notably increased *in-vitro*. CAR-T cells Secrete Inflammatory factors like IFN-γ and GM-CSF, which increase CD80/86 expression and probably induce M1 polarization of CAR-Ms. Upregulated CD80/86 on CAR-Ms may ameliorate CAR-T cell activation and fitness	[130]
**Lung cancer brain metastasis cell (H2030BrM)**	-	Anti-MSLN CAR-M with MyD88 signaling molecule	In the humanized mouse model, CAR-Ms penetrated BBB and significantly reduced brain metastasis growth. The CAR-Ms exhibit antigen-specific phagocytosis activity against MSLN-positive tumor cells. Also, CAR-M demonstrates much fewer neuron toxicities and liver compared to CAR-T	[181]
**Mammary gland squamous carcinoma cell line (HCC-1806)**	-	RAW 264.7 derived Anti-MSLN CAR-M with TLR4 and TLR2-based toll-like receptor signaling domains	*In-vitro*, MOTO-CARs effectively kill cancer cells and secrete notable levels of TNF-α, which displays that MOTO-CARs polarize toward the M1 phenotype upon target recognition *In-vivo,* the MOTO-CARs can traffic effectively to the tumor site and substantially reduce tumor burden compared to the mock control	[182]
**Non-small cell lung carcinoma NCI-H460 and A549 cell lines**	lentiviral or adenoviral (Ad5f35)	Human monocyte-derived anti-TK1 CAR M with TIR signaling	*In-vitro*, after transduction, MOTO-CARs exhibit sustainable M1 phenotype and express low levels of CD163 and high levels of CD14, CD80, and CD206. MOTO-CARs demonstrated a nearly fourfold increase in killing activity against cancer cells compared with the controls	[183]
**HT1080** **cells**	Lentiviral	CB-HSPCs, THP-1 and MONO-MAC-6 anti-CEA CAR-M with different intracellular domains including 2B4-DAP12, CD28-CD3ζ	Ex vivo, the CAR gene was transduced to CB-derived HSPCs successfully and generated CAR-Ms have stable CAR expression. Also, any evidence of that CAR expression might alter CD34 positive cell-derived macrophage morphology, phenotype, or basic anti-bacterial phagocytic function, but it was not observed. Both anti-CEA-CARs showed antigen-specific function by increased cytokine secretion, and CD3ζ CAR-expressingTHP-1-derived macrophages showed enhanced antigen-specific phagocytosis of target cells	[143]

*Abbreviations*:* TK1* Anti-Thymidine Kinase1, *hPSCs* Human pluripotent stem cells, *GD2* Disialoganglioside, *iPSCs* Induced pluripotent stem cells, *MUC1* Mucin short variant S1, *TNFα* Tumor necrosis factor, *HER2* Human epidermal growth factor receptor 2, *ECM* Extracellular matrix, *PBMC* Peripheral blood mononuclear cells, *ALK* Anaplastic lymphoma kinase, MPEI, *NP-pCAR* Nano particle-plasmid chimeric antigen receptor, Epidermal Growth Factor Receptor (EGFRvIII), *TLR* Toll-like receptors, *MerTK* MER Proto-Oncogene, Tyrosine Kinase, *Megf10* Multiple EGF-like-domains 10, *FcRγ* Fc receptor γ-chain, *Bai1* Brain angiogenesis inhibitor 1, *MHC-I* major histocompatibility complex, *TME* Tumor microenvironment, *NK* Natural killer, *DC* Dendritic cell, *TAA* Tumor associated antigen, *PI3K* Phosphatidylinositol-3 kinase, *MIP-2* Macrophage inflammatory protein 2, *MCP-1* Monocyte chemoattractant protein-1, *CXCL1* CXC motif chemokine ligand 1, *GM-CSF* Granulocyte macrophage colony-stimulating factor, *MSLN* Mesothelin, *BBB* Blood brain barrier, *MyD88* Myeloid differentiation primary response protein, *CB* Cord blood, *CEA* Carcinoembryonic antigen, *DAP12* DNAX activating protein of 12 kDa

**Table 4:** Human studies CAR M-based cells

NCT number	Tumors	Phase	Macrophage source	Study type	Gene transfer	Initial year
**03608618**	Advanced ovarian and peritoneal mesothelioma	I	PBMCs	Clinical trial	mRNA transfection	2018
**04660929**	HER2 overexpression solid tumors	I	Primary human macrophages	Clinical trial	Adenovirus transfection	2020
**05007379**	Against organoids from early and advanced breast cancer patients	I	N/A	Cohort	N/A	2021
**05138458**	Refractory/ relapsed T-cell lymphoma	I/II	MT-101 (Gathered from PBMCs)	Clinical trial	mRNA transfection	2021

The studies showed CAR M cells have sustainable M1 phenotype, secreted proinflammatory factors, and polarized M2 macrophage toward the M1 phenotype [105]. Also, it has been demonstrated that the CAR M anti-cancer action does not relate to the M1/M2 states induced via IFNγ, LPS, and IL-4 [121]. Alao about the iPSC-derived CAR M, Zhang et al. showed that the CAR-iMac polarization state is closer to M2 in the absence of antigen however in the presence of antigen the CAR iMacs tend towards M1 phenotype. So, they targeted K562 cells with expressing CD19 or K562 cells and mesothelin-expressing OVCAR3 ovarian cancer cells *in-vitro* and saw in the presence of antigen-bearing cancer cells promotes CAR-iMacs pro-inflammatory cytokines expression and phagocytosis in an antigen-dependent manner and polarize them toward an M1 state [129]. This is likely because not all the CAR-iMacs retain the same polarized phenotype in the TME. Still, their functions need to be enhanced by modifying CAR-iMacs to have a durable M1 phenotype or designing a more suitable CAR [129].

Regarding CAR M action, the investigations exhibited variable anti-tumor effects by different intracellular domains. For example, a study developed anti-CCR7 CAR M by lentiviral transducing of anti-CCR7 CAR into macrophages. Anti-CCR7 CAR contains intracellular signaling domains derived from TLR2, TLR4, TLR6, MerTK, or 4-1BB-CD3ζ. Among these CARs, CAR M with MerTK intracellular domain exhibits the most phagocytic and anti-tumor activity against tumor cells. MerTK CAR M reduces tumor burden, increases median survival time, and creates an inflammatory environment by increasing the levels of proinflammatory cytokines in serum, such as IL-β, IL-6, TNF-α, and monocyte chemotactic protein (MCP)-1 *In-vivo*. MerTK CAR M displays targeted anti-tumor function in both *in-vitro* and *in-vivo* [131]. Also, Morrissey et al. designed a new type of CAR called CAR for phagocytosis (CAR-Ps). The CAR-P structure consists of CD19 targeting ScFv, the CD8 transmembrane domain, and different intracellular domains, including Megf10, FcRγ, Bai1, and MerTK. CAR M with the Megf10 or FcRγ intracellular domains demonstrates the greater phagocytic capacity of CD19^+^ cells compared to Bai1 and MerTK. The CD3ζ subunit of the TCR can trigger phagocytosis of CD19^+^ cells to a comparable level as the Megf10 intracellular domain. Trogocytosis, through nibbles of other cells, was more common than whole-cell phagocytosis, suggesting that CAR-dependent phagocytosis signaling was insufficient to trigger whole-cell phagocytosis. Therefore, they generate ‘tandem’ CAR by fusion of the portion of the CD19 cytoplasmic domain that recruits the p85 subunit of PI3K to the CAR-P-FcRγ. Also, CAR-P containing PI3K intracellular domain alone could induce whole cell phagocytosis more than the CAR-P-FcRγ. In addition, CAR-P tandem macrophages notably decreased the number of tumor cells and showed much more whole-cell phagocytosis capacity than the CAR-P-FcRγ; however, both exhibited similar potency at destroying tumor cells [106]. Furthermore, the TLR showed more pro-inflammatory activity and killing persistence comparison CD3ζ, and when these receptors combined, they increased anti-tumor activity and contributed to retaining M1 polarization of CAR-iMacs. The CD3ζ-TIR-CAR-iMACs showed more substantial anti-tumor capability than CD3ζ-CAR-iMACs and TIR-CAR-iMacs *in-vivo* [128].

In addition to the high cytotoxic effects of CAR M cells, they have a lesser possibility of creating adverse effects. One study showed CRS-related cytokine increase only 2- to fourfold in CAR M compared to a 30- to 8,000-fold increase in CAR T cells, demonstrating that CAR M has lesser CRS risk than CAR-T cells [121]. The CAR M cell studies have shown no sign of systemic toxicity [123–125]. Although, in a study, the researcher utilizes CAR M (MerTK)-mediated adoptive cell therapy, cutaneous and intestinal toxicity in the high dose of CAR M cells was observed [131].

So, based on the CAR M preclinical, CAR M could have promising results in the studies and create good opportunistic for clinical studies. The CAR M cells can have an optimistic outcome in tumor elimination due to altering the TME, increasing immune cell infiltration, and eliminating targeted tumor cells. Also, CAR M can utilize different extra and intracellular domains for better function while lowering side effects outbreak. By the way, more study is needed to evaluate CAR M cells' efficacy and safety.

### Clinical application of CAR macrophage cells

To date, there are four clinical studies of CAR M cells, and none have been published yet, so the data was gathered from the Clinicaltrials.gov site. But recently, one article mentioned two new studies (NCT04405778 and NCT05164666) that worked on the CAR M cells, while more data require approval to determine whether they are related to CAR M [184].

The first study was developed by MaxCyte Company and evaluated the drug by the name MCY-M11 (NCT03608618). MCY-M11 was designed by delivery of mRNA into PBMCs to express anti-mesothelin-CAR and utilized in patients with advanced ovarian cancer and peritoneal mesothelioma. In phase I, they worked on the dosage augmentation to investigate engineered cells' safety, feasibility, and tolerability and infused them as three intraperitoneal injections per week. Patients were the women with high-grade serous adenocarcinoma in the ovary, fallopian tube, or primary peritoneum that have resistance to platinum-based chemotherapy before, and the patients with peritoneal mesothelioma who had recurrence after prior chemotherapy. The trial will also evaluate multiple courses of treatment and the used preconditioning drug with cyclophosphamide. The preliminary results of this study showed that CAR manufacturing happened in less than one day. The patients were divided into several groups based on the dose-receiving to DL1 1.0 × 107, DL2 5.0 × 107, DL3 1.0 × 108, DL4 5.0 × 108 cells/dose and three doses in week infusion as intraperitoneal without any preconditioning therapy. The primary results showed that the DL1 and DL2 received the drugs without any side effects, and 11 patients in the DL1, DL2, and DL3 have safety infusion and tolerance. As a result, no infusion-related or limiting side effects were present, and neurotoxicity was present. Most adverse effects are grade 1–2 and mild, except one patient in DL3 had grade 2 pericarditis, transient neutropenia, and fever. Indeed, one patient in DL2 had confusion, and another in DL3 had experienced an enterocutaneous fistula. Any report about the death or discontinuations related to the treatment had not existence.

Regarding the efficacy, three patients in the DL2 had stable diseases by RISTRICT 1.1. Among them, one patient did not continue the treatment, one followed for six months and had long-lasting conditions, and the last one had stable diseases for two months. In DL3, only one patient had stable disease for two months; the other enrollment is ongoing. So, the feasibility of MYC-M11 for 1-day production and intraperitoneal delivery showed that the patients experienced stable diseases after one-cycle of treatment, but the final results not be published [185].

The second study was the first-in-human, open-label study of CAR-M in HER2-overexpressing solid tumors that Carisma Therapeutics developed in phase I. Anti-HER2 CAR M (CT-0508) engineered with chimeric adenoviral vector Ad5f35 and administrated to 18 patients with relapsed/refractory tumors with over-expression of HER2. The primary results indicators encompass the safety and tolerability of CT-0508 alone and combined with pembrolizumab (anti-PD-1 antibody) by estimating the frequency, the feasibility of manufacturing, and severity of adverse events such as CRS (NCT04660929). In the preliminary results, the seven patients with various solid tumors (breast [2], esophageal [2], cholangiocarcinoma, ovarian, and parotid gland tumors) were treated with CT-0508. The CAR M production was successfully done successfully with high purity and suitable CAR expression. Also, the infusion was tolerable with minimum adverse effects (grade 1–2 of CRS and grade 2 infusion reaction) that were managed without needed tocilizumab. Also, the treatment did not reveal any end organ damage or on target-off tumor toxicity. The three patients reached stable diseases and one had progression diseases among patients who arrived at eight weeks. The CT-0508 could extravasate from blood fast, accelerate myeloid cells activation, and enhance T cell actions like proliferation, activation, and infiltration, so the drug improved tumor-infiltrated T cell expansion. The CT-0508 could exhibit promising results in solid tumor treatment [186].

Another observational study was developed by the Centre Oscar Lambret with CAR M (CARMA-2101) but not yet recruited. This study is a cohort study to determine the CAR M anti-tumor features against the patients with breast cancer-derived organoids who need surgery or a tumor biopsy as part of their care. Other biological samples will be collected from blood to analyze the host’s inflammatory status. For primary results from indicators, the anti-tumor function of the CAR-M is evaluated against organoids from HER2^−^, HER2-low, and HER2^+^ breast cancers for two years and will be compared with non-modified macrophages. As a secondary results indicator, the anti-tumor activity of the CAR-M is evaluated against organoids from patients with early and advanced breast cancer for two years (NCT05007379).

The last clinical trial is a phase I/II, open-label, multiple ascending dose multicenter study of MT-101 in patients with CD5^+^ relapsed/refractory T cell lymphoma. MT-101 is generated from the engineering of myeloid cells obtained from the patient's blood and subsequently administered intravenously (IV) back into the patient's body. This study has two sections; the first will consist of four groups of participants. Cohorts 1 and 2 will get a modest dose of MT-101 biological (CD5 AKAT cells), while cohorts 3 and 4 will get a higher dose of cells to evaluate safety and tolerability. In the second section of the study, depending on the outcomes of the first section, cells with or without chemotherapy (Fludarabine and Cyclophosphamide as IV infusion) will be given to patients. It will evaluate the safety, tolerability, and effectiveness of MT-101. Over three weeks, the drug product will be infused into all patient groups six times. Based on observed adverse events, the primary result indicators are safety, tolerability, and any possible dose-limiting toxicities. Secondary results encompass MT-101 cell kinetics in blood and the objective response rate (NCT05138458).

### Challenges in CAR macrophage therapy

Like other therapy methods, CAR M therapy has some challenges in production and treatment aspects. These challenges begin with manufacturing CAR M cells and continue until administration. Unlike other immune cells, macrophage cells have a lower desire to circulate in the blood and cannot expand like T cells, so collecting and developing these cells is a huge problem in CAR M production [9, 58]. Also, the macrophage cell in previous research has limited sources and little experience working with new sources. While the *in-vivo* genome inducing has particular challenges and needs more investigations in humans.

Moreover, the heterogenicity of human macrophages compared to mouse macrophages and limited knowledge about humans are other dilemmas in CAR M production *in-vivo* [187]. In addition, the clonal diversity of the tumor as a cancer response against treatment is an essential factor for tumor growth and metastasis that decreases the anti-cancer treatment functions [188]. Indeed, finding the best vector for genetic modification is an arduous choice in CAR M manufacturing. The previous studies sometimes used viral transfection, but the macrophage cells have a remarkable resistance to viral transfection and may induce insertional mutation into the cell genome [189]. So, these CAR M problems may require repeat doses to sufficient cells for anti-cancer action [156].

Furthermore, chemotherapy as an approved combination therapy with previous treatment in cancer patients could hurt macrophage-based immunotherapy. Therefore, the combination systems must be chosen carefully at an earlier treatment line [189]. The safety of CAR M in humans has not been approved and requires more investigation. Still, it’s expected that some side effects, because of the natural features of macrophage cells, happen in this therapy, like GVHD. But about particular CAR T cell adverse effects like CRS, HLH/MAS, or GVHD, maybe the CAR M cell has lower worry. However, the manufacturing of CAR M cells is based on the M1 phenotype for anti-cancer specialists. Still, in overactivation of the cell, they release the IL-1 and 6, so they can mainspring the CRS responses [189]. The CAR M has some challenges in the solid tumors field due to the unique features of these tumors and complex TME. Although the CAR M had superior performance in the solid tumor models, they have various past problems like tumor heterogeneity, antigen escape, the suppressive effect of TME, and cell exhaustion. Besides, the CAR M had good potential in animal cancer models, but the human cancer TMEs have a more complicated structure than animal models [190, 191].

Indeed, the effective persistence and trafficking of CAR M cells in solid tumors is a significant challenge and an improved homing by some migratory molecules like CSF-1 receptor tyrosine kinase, integrins, the integrin co-activator kindlin-3, and receptor-like protein tyrosine phosphatase ε, MMP10 and αMβ2 and αDβ2 [192–195]. The CAR M also has some problems administering exogenous cells because the infused cells across the lung and into the liver. Hence, the drug delivery of CAR M in tumor sites is another challenge in this therapy [187]. Furthermore, the mechanism of resistance to the CAR M therapy has not been discovered, so this would be a big problem in recurrent/resistant tumors to CAR M cell therapy. As the last challenge, it is natural that clinicians encounter new treatment methods and would be some worries about the efficacy, *in-vivo* required dosing, side effects, cost, and recurrence of tumor, but with the progression in studies and using different novel solutions, this worries can be low and accepted as a promising treatment method.

## Solutions

### Strategies for improvement of CAR function, control, and safety, novel CAR structures, new CAR generations, and gene-engineering

One of the essential and valuable strategies for improving CAR M cells is to elevate the function of the CAR structure by utilizing various generations or types of CARs. Today, a novel generation of CARs has been introduced. CAR M will overcome fundamental barriers like expansion and production by using the new generations, especially the fourth or fifth generation [196]. Indeed, new types of CARs can help in problems like antigen escape, adverse effects, *in-vivo* survival, and exhaustion. Utilizing multi-specific CAR like bicistronic CAR, Tan-CAR, and loop-CAR, which target more than one antigen, can help against the antigen escape process [197–201]. The specificity of CAR-armored cells could be increased by employing Logic Gated CARs such as split-recognition CAR, inhibitory CAR, SUPRA-CAR, and SynNotch CAR. Split-recognition CAR consists of two CAR structures. One structure contains the primary signaling domain, and the other has co-stimulatory domains, so activating the armored cell requires stimulation of both structures. Inhibitory CARs (iCARs) also consist of two CAR structures; one contains an inhibitory intracellular domain, and the other includes a stimulatory intracellular domain; thus, CAR cells stay inactive until off-target antigens exist [202, 203]. In addition, due to the different glycosylation patterns of cancer cells compared to normal cells, we can improve CAR specificity for target antigens and reduce on target-off tumor toxicity by designing glycan-targeting CARs (sweet CARs). Different ABD can also target glycans, including ScFv, lectin, or intelligent anti-glycan reagent (SAGR) [204]. For example, McKenna et al. developed CAR T cells with modified lection (H84T BanLec) as ABD of CAR. H84T CAR T cells showed strong anti-tumor capacity against PDAC *in-vitro* and *in-vivo*. Also, any evidence of side effects was not observed [205].

Indeed, for improvement and control, CAR activity and adverse effects can use various technologies like suicide genes, elimination genes, and targeted activation. Integrating suicide genes allows selective depletion of the CAR-armored cells and can prevent the progression of side effects, especially the on target-off tumor. This technology is brilliant in CAR T cells due to some fetal adverse effects but usable in CAR M because of the same side effects. Also, dimerized death molecules like Caspase 9 and Fas can cause selective depletion, too [206]. In this method, the CAR contains the FK506-binding proteins and activates them to start the downstream caspase by initiating the apoptotic pathways. These suicide genes were used widely in preclinical and clinical studies. They achieved significant results in controlling CAR T cell activation, as one single dose of small-molecule dimerizing agents (AP1903) can delete 90% of ICASP9 T cells in 30 min [207].

Furthermore, the use of elimination genes can help to control CAR. In this method, the cell is modified to express cell-surface antigens like EGFR or CD20 that, with infusion, the associated mAbs like rituximab or cetuximab can stimulate cell death [208, 209]. In a vast preclinical study, the research compares various safety switch technologies and gets results that when the IC9 and rituximab combine, the best results have been achieved [210]. However, due to prior heavy treatments and connecting the mAbs to normal tissue of the body, the progression of this method has some barriers and needs more investigation. Indeed, as we said, dual-targeting CARs like split-recognition CAR can also help CAR control and reduce the on-target-off tumor side effects in the patient’s body [211]. Multiplex drug-gated CARs were recently introduced to improve control and increase CAR safety, like versatile protease-regulatable CARs (VIPER) [212].

### Combination therapies

#### Conventional therapies like chemotherapy and radiotherapy

Chemotherapy and radiotherapy (RT), as conventional cancer therapies, can potentially enhance cell functions against tumor cells and seem good options for combination therapy with CAR M cells but require more caution [213].

Regarding chemotherapy, it's predicted that the chemotherapeutic agents can help the macrophage-based therapies by changing the macrophage phenotype to anti-tumor types and increasing their recruitment to TME [95]. A past study used chemotherapy agents for TAM depletion. It achieved anti-cancer effects, but this method can have binary effects on the CAR M-based therapy as beneficial or detrimental to macrophage phenotype and recruitment. TAM-depletion for cancer treatment is a rational way, but when this treatment is combined with CAR M, it may have harmful effects on the *in-vivo* production and treatment pathway [95]. So, if the researcher wants to use chemotherapy with CAR M therapy, they must take the treatment toward depletion of M2-like macrophage and augmentation of M1-phenotypes. Therefore, CAR M may enhance the cancer response to chemotherapy and prevent chemo-resistance by shifting M2 macrophage toward the M1 phenotype.

About RT, it seems that a good combination occurs between CAR M and RT in cancer patients. RT can achieve promising results in CAR M-based therapy by the effect on the recruitment of macrophage cells in tumor sites and polarization. By altering the extracellular matrix and inducing the secretion of soluble factors like cytokines, ILs, and chemokines, RT technology can increase macrophage recruitment in TME [214]. Indeed, RT could modulate macrophage polarization to treatment favor. For example, radiotherapy could enrich the TME with M1-like macrophages and decrease the M2-like of them [215]. It is imperative that attention to the binary effect of RT on macrophage function and for the final indication of use, it needs more investigation because RT, like chemotherapeutic agents, can have advantageous/disadvantageous effects on macrophage-based therapy. In conclude, the RT may sensitize CAR M cells via the abscopal effect and induce CAR M cell migration into non-irradiated tumor sites, which may be linked to the increase of intra-tumoral cytokines and chemokines, the release of neoantigens, and endogenous immune cell activation [216–218].

#### Oncolytic virus

One of the options for combination CAR M therapy is oncolytic viruses (OV). Due to the mechanism-based selectively, this approach eliminates cancer cells, and acceptance of the other additional therapeutic agents is a desirable treatment method [219]. However, due to the anti-viral features of macrophages, the interaction between OVs and macrophages is complicated in combination therapy. The old approaches suggested the depletion of TAMs by various methods like chemotherapy drugs, but new techniques showed that combining them can have brilliant therapy results [220, 221]. The correlation of OVs and macrophage cells seems to induce the TAM re-education of them; for instance, in one study, the delivery of CCL-16 by adenoviruses in a mixture with CpG caused the switch of M2 macrophages to M1 phenotype and increased anti-tumor responses [222].

Furthermore, the armored OVs by genes that give the cytokines secretion ability to them can help re-education TAMs [223]. Indeed, the TAMs are a promising vehicle for drug delivery in tumor sites; one study derived a mathematical model for infiltration of tumor spheroid with the ability to release oncolytic adenoviruses under hypoxic conditions by macrophage cell transportation. This model predicted that this cell combination with radiotherapy, when used immediately after radiotherapy, is a promising approach for coordinating the maximum therapeutic efficacy [224]. Other studies have shown that the TAMs have essential roles in supporting the tumoricidal effect of the OV (HSV1716) [225].

The other studies showed the synergistic effect of OVs and macrophage therapy in colorectal, glioblastoma, breast cancer, pancreatic cancer, and neuroblastoma [226]. Indeed, the combination of an oncolytic virus with CAR-armored cells has been examined in CAR T cell fields. Several pre-clinical studies demonstrated that CAR-T cell and oncolytic virus combination therapy reduce tumor growth and increase survival [227]. For example, Watanabe et al. utilized an adenoviral oncolytic virus that produces IL-2 or TNFα in combination with CAR-T cell therapy. The combination therapy was shown to decrease cancer metastasis, increase recruitment of T cells (both CAR T cell and host T cell), polarize macrophages toward the M1 state, and facilitate DCs maturation in pancreatic ductal adenocarcinoma (PDA) models [228]. Therefore, the combination of CAR M cells with OVs may enhance CAR M therapy, especially in Turing cold tumors to hot tumors, by accelerating tumor milieu altering toward anti-tumoral, increasing T cells and NK cells trafficking, and promoting the bystander macrophages shifting toward anti-tumoral phenotype and enhancing antigen presentation process.

#### Combination with other brothers, CAR T or CAR NK cells

Combining various CAR-armored cells in one therapeutic approach cloud is a suitable method to improve treatment efficacy. For example, Liu et al. demonstrated that using CAR-M FcRγ and CAR-T cells together has substantially greater cytotoxic capacity than CAR M or CAR T cells alone *in-vitro*. In addition, inflammatory factors such as IL-1β, IL-6, IFN-γ, CXCL1, MCP-1, and MIP-2 have notably increased in CAR M and CAR T cells combination. CAR T cells secrete inflammatory factors like IFN-γ and GM-CSF, which increase CD80/86 expression and probably induce M1 polarization of CAR Ms. Unregulated CD80/86 on CAR M may ameliorate CAR T cell activation and fitness. [130]. Combining CAR NK with CAR M cells can improve the treatment efficacy by covering their gaps. For example, the CAR M can eliminate the tumor cells by brilliantly infiltrating the tumor. In contrast, the CAR NK cells can regulate the other compartment of the immune system and create a synergistic treatment. Also, maybe the CAR NK by secretion of IFN-γ can synergistically alter the TME metabolic fitness. Indeed, the CAR M boosts the CAR NK infiltration into TME and improves tumor elimination. By the way, this combination has not been done, so maybe publishing more information. This combination method, due to the manipulating of immune systems, seems appropriate for solid tumors with poor prognosis and aggressive such as glioblastoma and pancreatic cancer in the end stages.

#### Combination with monoclonal-antibodies

CAR M ability to eliminate cancer cells can be enhanced via mAbs such as anti-CD47 and anti-PD1. Pierini et al. exhibited that utilization of anti-HER2 CAR M in combination with anti-PD1 alters TME, reduces tumor growth, and increases overall survival more than CAR M or anti-PD1 monotherapy [180]. Also, in another study, researchers utilized FcRɣ CAR M in combination with anti-CD47 antibodies against Raji cells and showed a 2.5-fold increase in whole-cell phagocytosis compared to FcRɣ CAR-Ms alone [106]. In addition, researchers developed plasmid CAR (pCAR) containing nano porters (NP), which target TAMs and generate CAR-Ms *in-vivo* and demonstrated that the combination of NP-pCAR and anti-CD47 showed the most potent anti-tumor capacity compared to NP-pCAR or anti-CD47 alone. A combination of NP-pCAR and anti-CD47 boosts the immune system response against tumor cells and enhances memory T cells and TILs recruitment in the TME, inhibiting tumor relapse without significant toxicity [229]. In another study, a combination of anti-PD1 with CAR M demonstrated further reprogramming of TME and tumor control [115].

Furthermore, several studies designed CAR-armored cells with the ability to secretion of antibodies; for instance, Suarez et al. designed anti-CAIX CAR T cells that secrete anti-PD-L1 antibodies and demonstrated that they can reduce T cell exhaustion recruited NK cells and further diminish tumor size in a mouse model of renal cell carcinoma [230]. In addition, Thakur et al. generated CAR-T cells without the extracellular ScFv domain, which are called “Headless CAR T cells” (hCART). They utilized hCAR-T cells in combination with HER2 or EGFR bispecific antibodies (BiAbs). They demonstrated that hCAR-T armed with BiAbs outperforms non-genetically engineered T cells armed with BiAbs, induces more significant cytotoxicity levels, and continues eliminating tumor cells under hypoxic conditions *in-vitro* [231].In summary, the CAR M plus mAbs boost anti-tumor treatment efficacy and prolong overall survival.

#### Combination with epigenetic drugs

Epigenetic strategies could be combined with CAR-armored cells to enhance the efficacy of treatment [232]. For instance, drugs, mainly those that can boost histone acetylation and/or DNA demethylation, can increase the expression of MHC I and II, CD40, CD80, and TAAs [233–235]. Epidrugs like HDACis and DNMTis increase CTA abundance and can improve cancer cell identification [236–239]. Also, Kailayangiri S et al. demonstrate that using EZH2 inhibitors could increase the expression of GD2 on Ewing sarcoma cancer cells to improve CAR-T cell's anti-cancer capacity [240]. In addition, promoting tumor suppressor miRs, such as miR-448 and miR-153, or inhibiting the expression of oncomiRs, including miR-155 and miR-21, could be potential therapeutic targets in combination with CAR-armored cell therapy [232]. For example, Huang Q et al. showed that miR-153 overexpression in cancer cells through inhibiting of Indoleamine 2, 3-dioxygenase one expression could promote CAR T cell killing ability *in-vitro* and suppress cancer progression in a murine colorectal cancer xenograft model [241]. In the results, the epidrugs may create suitable TME for CAR M cell activity, enhance their ability to recognize tumor cells, and reduce antigen escape and modify TME.

### Improve persistence and migration of CAR M cells

One crucial factor affecting the CAR armored cell therapy efficacy is their persistence and migration ability. The common strategies that can improve the persistence and migration of CAR armored cells include utilizing the novel CAR structures, engineering them to express chemokine receptors or cytokines, targeting immune modulatory markers, and combining them with other treatment methods like OVs [242]. For instance, in a preclinical study, researchers designed anti-Aβ CAR-Ms with FcRγ intracellular domain to target and resorb amyloid plaques. Also, CAR-M cells' persistence and expansion ability were increased by engineering them to secrete M-CSF, demonstrating that M-CSF secreting CAR-Ms can lower amyloid plaque load near the injected area [146]. Also, targeting PD-1 is another method to increase CAR armored cell persistence. Until now, the anti-PD1 is the only common ICI utilized with CAR T, CAR NK cells, and CAR M cells, demonstrating superior efficacy compared to monotherapy [243–245]. In addition, various candidate has been found that could improve the CAR M migration, like receptor-like protein tyrosine phosphatase ε (RPTPε), metalloproteinase 10 (MMP10), the integrin co-activator Kindlin-3, CSF-1 receptor tyrosine kinase, and αMβ2 and αDβ2 integrins [193, 194, 246, 247]. On the other hand, the controlling CAR M persistence in tumor sites can be utilized from apoptotic molecules with caution. It was so, using anti-apoptotic factors to create novel CAR M structures like TGFβ-activated kinase (TAK1) activators, TAK1-binding protein 1 (TAB1), Mcl-1, Bcl-Xl, and TAK1-binding protein 2 (TAB2) [248, 249]. Furthermore, it is essential to consider that these strategies differ to some extent among CAR armored cells due to different gene-expression profiles among T cells, NK cells, and macrophages.

### CRISPR-cas9 and Cas-CLOVER gene engineering

Macrophage engineering is one of the significant challenges in CAR M production and action, so using various approaches to facilitate these challenges would be very valuable. The CRISPR/cas9 approach is one of the vital methods utilized widely in cell immunotherapy and has shown distinguished results. In the CAR M cell therapy, the CRISPR/cas9 was used for CAR gene knock-in and had brilliant results in this manner. For example, in a study, the researcher utilized CRISPR/cas9 to integrate the anti-GD2 CAR genome into the AAVS1 locus of hPSCs. These CAR M cells could eliminate the GD2-expressing neuroblastoma *in-vitro*, in-vivo, and melanoma *in-vitro* [121]. Indeed, the CRISPR/cas9 technology can improve the CAR M function by targeting different axes in the macrophage cells. For example, targeting the aconitate decarboxylase 1 (ACOD1) Kelch-like ECH-associated protein 1(KEAP1) as essential regulators in the pro-inflammatory state of macrophage and with CRISPR and knocking out them created kind of the CAR M cells with more persistence and stronger polarization, more ROS production, and high potent phagocytosis and cytotoxic *in-vitro* study. The CAR M cells with depletion of ACOD1 with CRISPR/cas9 could show high anti-cancer function in ovarian and pancreatic mouse models and improve their life span.

Furthermore, combining this structure with ICIs had synergistic effects [250]. In another study, depletion of the SIRPα as a “don’t eat me” signal with CRISPR/cas9 in CAR M cells showed synergism efficacy in the anti-tumor field. At the same time, the SIRPα knock-out alone had failed in increasing anti-cancer macrophage responses [251, 252].

In addition, knocking out some agents that precipitate in M2 phenotype polarization and promote tumor survival have been known to be targeted in macrophage but not used in CAR M manner; for example these factors like kindlin2, osteopontin, lysosome-associated membrane protein type 2A (LAMP2a), IL-8, and tumor-secreted protein S (Pros1) [253]. Except for CRISPR/cas9, other CRISPR-based technology was used to regulate the gene transcription, like chromatin remodeling factors and catalytically dead Cas9 (dCas9) [254]. For example, the study utilized CRISPR interference (CRISPRi) for silencing CD209, CD45, and TICAM1 genes in monocytes [255], or in the other ones, the dCas9 fused to the methylase for silencing HIF-1α to reduce TAM immunosuppressive action. The HIF-1α epigenetically repressed macrophage could help to reprogram tumor immunosuppressive microenvironment in murine melanoma models [256].

Also, we can develop more suitable CAR-armored cells by utilizing Cas-CLOVER to knock in or knock out genes. Cas-CLOVER, a site-specific nuclease, is a novel gene editing method with high efficiency and lower off-target activity than CRISPR-cas9. The Cas-CLOVER system consists of a dual gRNA-guided nuclease that incorporates a fusion protein made of a catalytically inactive Cas9 (dCas9) and the Clo51 endonuclease in each half-site subunit of the enzyme. Since the formation of a dimer is required for Clo51 activity, DNA cleavage is solely dependent on the concurrent on-target binding of two distinct gRNA-guided endonucleases in a particular vicinity [257]. For instance, Madison et al. developed allogenic CAR-T cells using the piggyBac transposon system to express CAR and Cas-CLOVER to knockout B2M and the TRBC genes that inactive MHC-I and TCR, respectively [258].

### Nano complexes

Several studies demonstrated the high potential of nano complexes to improve some CAR-M challenges like reduction cost, facilitation manufacturing process, and decreation tumorigenic risk in viral vector transduction. Kang et al. used mannose-conjugated poly ethylenimine (MPEI) as a gene delivery carrier to target macrophages and IFN-γ gene for CAR M polarization into M1 type, so created nano complexes of MPEI and CAR-IFN-γ pDNA (MPEI/pCAR-IFN-γ) *in-vivo*. Transfection with MPEI/pCAR-IFN-γ was insignificant (approximately 14 percent). Still, it could significantly alter the M2 phenotype to M1 and sustain the M1 state in macrophages even after phagocytosis of malignant cells *in-vitro*. The MPEI/pCAR-IFN-γ injected into Neuro-2a tumor-bearing mice notably halted tumor growth with no clue of systemic toxicity, decreased the Treg cell and increased activated CD8^+^ T cells in the tumors. Additionally, CAR-M enhanced the function of CD8^+^ T cells, likely by CAR-dependent tumor cell phagocytosis [120]. In another study, researchers generated anti-CD133 CAR-Ms *in-vivo* by intra-cavity injection of NP–hydrogel superstructure after resection of glioblastoma mass. NPs deliver the plasmid CAR (NP-pCAR) into macrophages existing in the glioblastoma resection cavity and create anti-CD133 CAR-Ms. NP-pCAR induces M1 polarization and increases the secretion of IL-1β and TNF-α of targeted macrophages *in-vitro*. The NP-pCAR could decrease the number of CD133^+^ tumor cells and cause tumor regression without traceable toxicity in the orthotropic mouse glioma model [147]. In addition, Gao et al. developed a synthetic DNA nanocarrier (P/PB/N/R nanoparticles) that consists of plasmid CAR with macrophage-specific CD68 or CMV promoter, NLS peptide, PBAE C32-122 polymer, and RP-182 peptide. They engineered brainstem gliomas TAMs through intratumoral injection of synthetic DNA nanocarriers. *In-vitro*, PCD68/PB/N/R nanoparticles engineered macrophages and produced CAR-Ms with M1 phenotype, greater phagocytic, and cytotoxic ability in an antigen-dependent manner. Intratumoral injection of P/PB/N/R nanoparticles generates anti-HER2 CAR-Ms by the engineering of TAMs and causes phagocyte of tumor cells, enhances the innate and adaptive immune system response, and tumor regression in mice without any notable side effects [121].

In another study, the researcher used an innovative gene delivery system named FDMCA that consists of different parts like 1,2-Dioleoyl-3-trimethylammonium-propane (DOTAP), Methoxy poly(ethylene glycol)-poly(lactide) (MPEG-PLA), and folic acid modified poly(ethylene glycol)-poly(ε-caprolactone) (FA-PEG-PCL). These FDMCA-nanoparticles loaded with macrophage inflammatory protein three β (MIP-3β), as an enhancer for anti-cancer, demonstrated up-regulated of MIP-3β in cancer cells and exhibited DC cell maturation, induced M1 phenotype, and activation lymphocytes. Indeed, the structure significantly helps tumor reduction and metastasis prevention in mice models [259]. Recently, one study used nanoparticles to deliver siRNA to silence CSF-1R. The results showed NPs had promising physiological features and a high level of selective uptake into the targeted macrophages and reprogramming to M1 phenotypes so they could improve apoptosis cancer cells. Indeed, this approach can utilize chemotherapeutic drugs as a co-delivery method for enhanced cancer therapy [260].

### Inhibition or switch of the M2 macrophages

As we saw in past parts, the M2-like macrophages have a promotion role in cancer progression. They are associated with poor prognosis cancer types, so the primary hypothesis is targeting these cells. These targeting methods can be utilized from various strategies to inhibit the M2-like in the TME and help cancer treatment. Several factors have been funded that have inhibitory or activatory effects on the M-like macrophage cells. Some activatory factors are monocarboxylic acid transporters, bone morphogenesis proteins, and chemokines like CXCL1, PI3k/Akt signaling pathway, stromal hyaluronan, and MiR-21/PDL-1. In other ways, some of the inhibitory factors are pseudomonal aeruginosa-mannose sensitive hemagglutinin, Cucurbitacin B, chemotherapy agents, IL-27, Dioscin, and mitomycin C/BCG [261–263].

By improving or suppressing these agents or shifting them to M1-phenotypes, we can interfere with CAR M cells and target the M2-like macrophages. For macrophage switch, several strategies help cancer therapy, for example, the use of micellar nano-drug targeting M2 peptide hidden in the OH-sheddable PEG for targeting cells in the acidic environment of TME. This structure could target the IKKβ siRNA and STAT6 inhibitor AS1517499, shift the M2 cell to M1, and suppress the tumor growth and metastasis with minimum side effects [264]. Indeed, the research targeted two nutrient transporters expressed in colon cancers: mannose receptors and secreted proteins acidic and rich in cysteine. This system can dually target the cancer cells and M2 macrophages, so they progress the mannosylated albumin nanoparticles with co-encapsulation of different drugs, i.e., disulfiram/copper complex (DSF/Cu) and regorafenib (Rego). As a result, the combination of DSF/Cu and Rego inhibits tumor growth in colon cancer with improved apoptosis, macrophage reeducation, and anti-angiogenesis [265].

In another study, the researcher used dioscin as a natural steroidal saponin for anti-cancer therapies. It demonstrated that it can induce macrophage transition, reduce M2 macrophages, and enhance phagocytosis [266]. Also, the studies showed that metformin could be used as a potent drug for M2 macrophage suppression, as it can suppress IL-13, trigger activation of AMPKα1, and block the M2-like polarization of macrophage so that it could inhibit the metastasis in Lewis lung cancer mouse models [267]. Furthermore, the all-trans retinoic acid (ATRA) can directly inhibit the M2-like macrophages to regulate cancer initiation in osteosarcoma models with a combination of IL-13 [268]. In one study, they have designed the nanoliposome for targeting M2-like macrophages by encapsulating ZA, hematoporphyrin monomethyl ether (HMME), and modifying M2pep peptide with the name M-H@lip-ZA nanoliposome. This structure could target M2-like macrophages appropriately and deplete them by functional remodeling of TME, like reducing the immunosuppressive function, elevating immune improvement cytokines, and intratumoral perfusion *in-vivo* and *in-vitro* [269]. In another approach, uses of miRs can restrain the M2-like cells such as miR-770, miR-183-5p, and miR-15b-5p [270, 271]. For instance, tumor-derived exosomal MiR-770 with downregulation of MAP3K1 can suppress the M2-like cells and inhibit the invasion in NSCLC cancers [272].

### Microwave and photothermal

Local ablative treatments, like microwave (MWA), can eradicate tumors through hyperthermic damage to cancer cells and trigger the release of immune-modulating substances such as danger signals, cancer antigens, and cytokines that trigger an immune response against the cancer cells. [273]. The MWA, as a non-aggressive method, can be used after or before CAR M therapy and may increase the treatment efficacy. The MWA was used in macrophage-based treatments and achieved significant results. For example, Cheng et al. utilized nanovesicles in combination with photothermal therapy. They designed hybrid nanovesicles (hGLV) that overexpress CD47 and load them with a photothermal agent (ICG). Hybrid nanovesicles and photothermal therapy combination methods promote DC maturation and improve the phagocytic capacity of macrophages by blocking the CD47-SIRPα axis between cancer cells and macrophages [274]. Indeed, the new photodynamic therapies (PDT) are used for cell targeting in the tumor site and could improve tumor elimination with different mechanisms and be completely safe. For example, in a study, the researcher utilized mannose-conjugated chlorin e6 (M-chlorin e6) and saw that M-chlorin e6 could target the M2-like cells that express mannose receptors, demonstrating a tumor growth depletion and TAM polarization [275]. Also, a recent study showed that combining AXL-CAR T cells with MWA compared to AXL-CAR T cell therapy alone has better anti-tumor efficacy in NSCLC patient-derived xenografts. Also, analyzed the phenotypes of macrophages demonstrated decreased M2 polarization in tumors [276].

## Conclusion

As a novel approach for solid cancer therapies, the CAR M cells are promising, with significant results and open new aspects of immunotherapy-based CAR structure. With excellent innate features and flexibility in accepting new gene engineering, these cells become the perfect candidates for immunotherapy. Although the last judgment requires more investigations, primary results in pre-clinical and *in-vitro* or *in-vivo* studies showed high efficacy and action against solid malignancies. With its distinguished function against cancers, the CAR M needed more experience in the dose required, side effects management, pretreatment conditions, post-treatment follows up, and other aspects of the treatment. The CAR M cell therapy, like other immunotherapy, requires new therapeutic tools for maximum efficacy, so experience in utilizing treatment methods is more needed. In recent years, the burgeoning of artificial intelligence (AI) has caused it to enter different fields, like medicine. Machine learning and deep learning as subsets of AI could help predict novel cancer-associated antigens, prognosis, and treatment response of CAR-cell therapies through medical images [277–279]. Also, AI could help improve the time-consuming and complexities of CAR cell production through automated CAR-T cell manufacturing [280]. Undoubtedly, the interaction between the immune system and genetic technologies can be an intelligent method against cancers and open new windows of cancer treatment.

### Supplementary Information

**Additional file 1.** Cover letter

## Data Availability

Data will be available upon request by the editor/reviewers.

## Abbreviations

TAM: Tumor-associated macrophage
NK cell: NATURAL killer cell
CAF: Cancer-associated fibroblast
TAN: Tumor-associated neutrophil
CXCR4: C-X-C motif chemokine receptor 4
CXCL12: C-X-C motif chemokine ligand 12
CSF-1R: Colony-stimulating factor 1 receptor
SIRPα: Signaling-regulatory alpha-protein
