# Supplementary material for: The CAR macrophage cells, a novel generation of chimeric antigen-based approach against solid tumors
Source: Biomark Res. 2023 Nov 28;11:103. doi: 10.1186/s40364-023-00537-x (PMC10685521; doi:10.1186/s40364-023-00537-x)
Supplement: Supplementary file 1 — Additional file 1. Cover letter [file 40364_2023_537_MOESM1_ESM.docx]

Cover letter

Aug 28, 2023

**---------------------------------------------------------------------------------------------------------------------**
The editorial department of Biomarker Research

Hi

Dear editor of the Biomarker Research Journal. I am submitting a novel review manuscript for consideration in your Journal entitled “The CAR macrophage cells, a novel generation of chimeric antigen-based approach against solid tumors” for publication in tour journal and benefit from your valuable comments. I confirm this work is novel, has not been published elsewhere, and has not been submitted to more than one journal for simultaneous consideration.
In advance, thank you very much for your consideration.
Yours sincerely,
Abdolreza Esmaeilzadeh
Department of Immunology and Cancer Gene Therapy Research
Center, Zanjan University of Medical Science, Zanjan, Iran.
Email address: a46reza@zums.ac.ir
Tel: +98-24-33140299; Fax: +98-24-33449553
Mobile: +98 9121414281

---------------------------------------------------------------------------------------------------------------------
